# Health of refugee children upon arrival in high-income countries: A scoping review

**DOI:** 10.1016/j.jmh.2025.100373

**Published:** 2025-10-29

**Authors:** Binyam Minuye Birhane, Angela Dawson, Andrew Hayen

**Affiliations:** a College of Health Sciences, Debre Tabor University, Debre Tabor, Ethiopia; b School of Public Health, University of Technology Sydney, Sydney, Australia

**Keywords:** Refugee children, Child health, Health status, Resettlement, High-income, Scoping review

## Abstract

**Background:**

Refugee children represent one of the most vulnerable population groups globally, facing higher health risks. This scoping review aimed to systematically identify, map, and synthesize recent evidence on the health outcomes of refugee children during the initial resettlement phases in high-income countries.

**Methods:**

We conducted a comprehensive search of major electronic databases, including MEDLINE, CINHAL, EMBASE, Emcare, Scopus, and Web of Science, to identify relevant peer-reviewed studies. The study selection processes adhered to the Preferred Reporting Items for Systematic Reviews and Meta-Analysis Extension for the Scoping Review guidelines. Furthermore, we applied the Joanna Briggs Institute (JBI) framework for scoping reviews to ensure methodological rigor and transparency.

**Results:**

Of the 3440 articles retrieved, we included 43 studies (38 cross-sectional, three longitudinal, and two cohort). Findings categorized into seven key thematic areas: (1) nutritional deficiencies (e.g., stunting, wasting, underweight, overweight, obesity, and vitamin deficiency); (2) infectious diseases (e.g., latent tuberculosis, hepatitis); (3) neglected tropical diseases (e.g., scabies and schistosomiasis); (4) dental caries; (5) Elevated blood lead level (6) inadequate immunization status; and (7) mental health problems.

**Conclusions:**

This review emphasizes that refugee children are resettled with a multiple burden of health problems. Comprehensive health screening and culturally responsive targeted interventions are essential in addressing unmet health needs of refugee children. Future research should focus on longitudinal study designs and the use of disaggregated data by countries of origin, age, migration pathways, resettlement context, and specific child health outcomes. Such approaches enhance understanding of health disparities, inform evidence-based health planning, and support the development of refugee health policies.

## Highlights

• Most of the included studies were cross-sectional; a few were longitudinal and cohort.
• Identified seven major health themes among refugee children upon resettlement in high-income countries.
• Nutritional deficiencies and infectious diseases were the leading health burden reported in the review.
• Neglected tropical diseases, elevated blood lead level, mental health issues, and poor immunisation coverage were also significant health problems.
• Findings revealed a need for targeted health interventions, programs, and inclusive polices to address unmet health needs of refugee children.

## 1 Background

By the end of 2023, >117.3 million people worldwide were forced to flee due to conflict, violence, persecution, or human rights violations, with >43.4 million identified as refugees. Children represent a significant proportion of this population, accounting for >41 % of refugees by 2022 (United Nations High Commissioner for Refugees (UNHCR), 2023a). In particular, between 2018 and 2022, >1.9 million children were born in refugee camps, highlighting the growing demographic of children living in displacement settings (United Nations Children’s Fund (UNICEF), 2023). Despite a higher burden of forced displacement, refugee resettlement in high-income countries has remained limited (United Nations High Commissioner for Refugees (UNHCR), 2023a).

According to the 1951 Refugee Convention and its 1967 protocol, a refugee is a person “…on protection from political or other forms of persecution, unable or unwilling to return their country of origin owing to a well-founded fear of being persecuted for reason of race, religion, nationality, membership of a particular social group, or political opinion” (UNHCR, 2001). The disproportionate impact of forced displacement, coupled with the challenges of growing up in refugee camps or precarious environments, highlights the need for targeted interventions and sustainable solutions for refugee children settled in high-income countries. Ensuring refugee children's health, well-being, development, and survival is not only a moral obligation, but also a global public health priority to create a healthy and productive future generation (Letourneau et al., 2013; Madewell et al., 2022).

The Sustainable Development Goals (SDGs), emphasizes the global imperative to ensure that all children, regardless of their background, have equitable access to high-quality health care, nutrition, and early childhood learning opportunities by 2030 (UNICEF, 2023; World Health Organization (WHO), 2018). However, children from refugee backgrounds face multiple risks that impact their survival and development and long-term wellbeing (Spencer et al., 2013). The experience of forced displacement is often marked by the cumulative multiphase health risks across the migration journey. These include exposure to conflict and violence, inadequate shelter, poor nutrition, limited access to healthcare services, heightened risk for infectious and endemic diseases, and prolonged stay in refugee camps (Cuadrado et al., 2023; Hauser et al., 2023).

Upon resettlement, these challenges are often exacerbated by systematic and structural barriers in refugee hosting countries, such as socioeconomic marginalization, discrimination, poor living standards, language and culture differences, and inadequate access to services, including healthcare, education, and social support systems (Orcutt et al., 2020; Spaas et al., 2022; WHO, 2022). These intersecting factors increase the risk of adverse physical, mental, social and emotional health outcomes and hinder the ability of refugee children to adjust and thrive in the new environment (Barthel et al., 2019; Dew et al., 2023; Hughes et al., 2017; Metzler et al., 2017).

Recognising and responding to the unique health needs of refugee children is therefore essential. These include minimizing barriers to health care and ensuring inclusive and equitable access to health care services that are responsive to the complex and evolving health needs of refugee populations (Chang et al., 2022). World Health Organization has implemented health and migration initiatives aimed at promoting the health and development of children. In addition to these, a call for universal healthcare coverage (UHC) that includes refugee populations, reflecting a global commitment to equity and inclusion in the healthcare systems, has been implemented. For instance, the WHO Department of Migration and Health released guidelines and an action plan to strengthen the health systems and improve the health of refugees (WHO, 2019, 2023a, 2023b). However, addressing the health needs of refugee children remains persistent and a lack of clarity in prioritizing refugee child health problems (WHO, 2022; Zimba and Gasparyan, 2023). One of the most critical barriers is the limited disaggregated population health data that captures their experiences and health outcomes of the refugee population. In many national health statistics, refugees are either not identified or considered altogether with other migrants, hindering efforts to monitor progress toward health-related Sustainable Development Goals (WHO, 2022). These data gaps limit the ability of policy makers, health systems, researchers, and international agencies to design and implement responsive and evidence-based targeted interventions, to ensure refugee children’s health needs are recognized, prioritised and adequately addressed (UNHCR, 2022).

While individual studies have documented health outcomes among refugee children across different high-income countries, existing systematic reviews have only synthesised evidence up to 2018, and often combined data from refugee and asylum seekers (Baauw et al., 2019). These may have contributed to a critical gap in capturing the recent burden of child health issues, as global displacement is increasing and the rise of refugee resettlement in high-income countries. Given the multifaceted health vulnerabilities and ongoing gaps in comprehensive health data, there is a pressing need for a synthesis of recent evidence on the health of refugee children. To address these gaps, we conducted a scoping review to explore current evidence on the health of refugee children following resettlement in high-income countries.

## 2 Materials and methods

### 2.1 Research questions

We developed a scoping review protocol according to the Preferred Reporting Items for Systematic Reviews and Meta-analysis Extension for Scoping Review (PRISMA-ScR) guidelines. The protocol is publicly available on the Open Science website (https://osf.io/tbpsf). The methodology was guided by the Joanna Briggs Institute (JBI) approach to the conduct of scoping reviews, which builds on the Arksey and O’Malley scoping review framework (Arksey and O'malley, 2005). Additionally, we adhered to the preferred reporting items using the PRISMA-ScR checklist and explanations to ensure methodological rigour and transparency, as detailed in Appendix 1 (Tricco et al., 2018).

We applied the population, concept, and context (PCC) frameworks (Peters et al., 2020) to formulate our review questions. The population of interest consisted of refugee children under 18 years of age. The concepts focused on the health status (morbidity, mortality), health needs, health problems, and health conditions (disorder or illness) of refugees upon arrival in high-income countries, as the WHO definition of health, “…as a state of complete physical, mental, and social well-being and not merely the absence of diseases or infirmity. Child health is a broad concept, and variation may exist in outcome measures and reporting across countries (Stein, 2024). Child health is defined as “…the extent to which an individual or group of children (birth to the age of 18 years) is able or enabled to a) develop and realise their potential, b) satisfy their needs, and c) develop the capacities that allow them to interact successfully with their biological, physical, and social environment” (NRCIM, 2004; Stein, 2024). We included child health measures (health conditions-disorder, morbidity, illness, mortality), functioning (manifestation of health-disability), and health potential (developmental potential).

In the context of this study, the term “upon arrival” refers to the initial periods following resettlement/establishment in refugee-hosting countries, during which the health of refugees is first assessed; however, no specific time frame, and this period differs across countries (Centers for Disease Control and Prevention (CDC), 2021; Chaves et al., 2016; Schrier et al., 2019). This could alternatively be stated as the post-arrival, initial resettlement, post-migration, and entry-level health screening phases.

Resettlement is defined as the process through which “refugees relocate to hosting countries that agreed to admit them with a legal status that ensures international protection and ultimately permanent residence.” Refugee-hosting countries provide them with legal and physical protection, including access to civil, political, social, health, economic, and cultural rights, similar to those enjoyed by nationals (UNHCR, 2023b).

This review was based on studies in high-income countries, as defined by the World Bank 2023 report of countries based on gross national income per capita (World Population Review (WPR) 2024). This scoping review aimed to synthesize and describe the available evidence on the health of refugee children during their resettlement phases in high-income countries.

### 2.2 Inclusion and exclusion criteria

We included studies published between 2013 and 2023 that explored the physical, mental, and overall well-being of refugee children (under 18 years old). Quantitative (cross-sectional, cohort, and longitudinal) studies were included in this review. We excluded articles without full text, case reports, case series, letters to editors, conference abstracts, studies that did not specify the age group of the participants, and studies that lacked specific information for refugee children (e.g. refugee population without disaggregated data, outcome not clearly stated, studies in the global context where extracting data specific to high-income countries was impossible)

### 2.3 Search strategy

A comprehensive search strategy was developed in consultation with the University of Technology Sydney research librarian. We conducted a comprehensive systematic literature search across multiple databases, including MEDLINE, EMBASE, Emcare, CINHAL, SCOPUS, and Web of Science, supplemented with a reference list of papers. We developed a search strategy using database-specific tools such as Medical Subject Headings (MeSH) for Medline and EMTREE for the EMBASE database. We used Boolean operators (AND, OR, and NOT) and truncation to refine our search strategy. We searched published articles from April 20 to July 30, 2023. Keywords for each concept were developed based on the literature search. The detailed search terms and full search strategy are provided in Appendix 2.

### 2.4 Screening and selection

We conducted a comprehensive search of electronic databases, search engines, and reference lists of included articles to identify relevant studies. Duplicate articles were removed using Covidence software, a systematic review management tool. Two reviewers screened titles, abstracts, and full texts. Discrepancies were resolved through discussion and consensus.

### 2.5 Data extraction

We developed a data extraction format to systematically extract relevant information from included studies. The form included authors, year of publication, study objective, study setting, participants’ country of origin, demographic characteristics (e.g., age), study design, sample size, and key findings (Table 1).

**Table 1:** Characteristics of the included studies.

Authors (Year)	Aim/Objective	Country of study	Target age group (0–18 years)	Participant's country of origin	Study design	Sample size	Key findings
(Shah et al., 2014)	To determine the nutritional status of newly arrived refugee children	USA	6 months-18 years	Bhutan Malaysia Thailand Africa Myanmar	Retrospective Cross-sectional	*N* = 555	Underweight • 17.2 % (92/534) overall; higher among refugees from Thailand; 8.7 % (15/172) in under-five children. Wasting • 12.2 % (21/172) among under-five children. Stunting • 18.6 % (22/172) among under-five children Anemia • 17.7 % of children were anaemic, more prevalent among African refugees, followed by refugees from Thailand; the highest in those under five, 21.8 % (40/183) and teenagers (12–15 years) Parasitic infection • 23.7 % had stool parasites (e.g., Giardia lambia (75 %), Dientamoeba fragilis (17 %), Trichuris trichiura (12 %), Hymenolepis nana (4.1 %), and Ascaris lumbricoids (4.1 %)); more prevalent among African refugees (34.6 %). Dental caries • 44.8 % (164/366) overall; more prevalent among refugees from Thailand (50.0 %) and Malaysia (45.3 %).
(Newman et al., 2019)	To assess the nutritional status of resettled paediatric refugees	Australia	2 months-18 years	South Asia Southeast Asia Africa Middle Eastern	Retrospective Cross-sectional	*N* = 1131	Underweight • 8.8 % (87/991) Overweight • 8.6 % (97/1124) overall; 5.3 % (7/133) in children under two years Obesity • 5.8 % (57/991). Stunting • 9.3 % (8/86) among children under two years of age Wasted • 2.3 % (31/133) among children under two years of age Anemia • 7.3 % and the highest among Southeast Asian children (11.4 % (41/359), and lowest in South Asian children 3.0 % (7/234); 12.8 % (47/368) among under-five children. Iron deficiency 12.3 % (47/368); 20.4 % (75/368) of under-five children are associated with prolonged breastfeeding (>12 months) and had inadequate dairy intake (41.0 %). Breastfeeding • Breastfeeding was sustained in 77.8 % (28/36) of infants <12 months and 44.9 % (44/98) of those 12–24 months; 27.1 % required formal dietetic follow-up Thalassaemia traits • 6.5 % overall; 31.1 % of these were anaemic Vitamin D deficiency • 50.3 % overall; most prevalent in Middle Eastern 60.1 % (140/233) and South Asian 59.8 % (140/234); 43.8 % (161/368) in under-five children Dental caries • 59.8 % (390/652) overall; 67.7 % in Southeast Asian children
(Dawson-Hahn et al., 2016)	To compare the nutritional status of refugee children with children from low-income families	USA	0–10 years	Somalia Iraq Myanmar	Comparative cross-sectional	*N* = 982	Overall malnutrition • 44.9 % had at least one form of malnutrition. Stunting • 20.1 % overall; 21.3 % (107/502) among under-five children. Wasting • 17.3 % overall; 14.3 % (72/502) among under-five children Overweight • 7.6 % • 8.6 % (43/502) among under-five years Obesity • 5.9 % overall; 6.2 % (31/502) among under-five children
(Walpole et al., 2018)	To assess the growth of children in a refugee setting	Greece	0–5 years	Syria Iraq	Cross-sectional	*N* = 109	Stunting • 17 % overall; 13 % were children under one year of age
(Grammatikopoulou et al., 2019)	To assess the malnutrition status of children in a refugee setting	Greece	1–18 years	Syria Afghanista	Cross-sectional	*N* = 192	• 13 % of children had at least one form of malnutrition, higher prevalence among females Underweight • 7.8 % overall; 3.1 % (2/65) among under-five children Stunting • 7.3 % overall; 9.2 % (6/65) among under-five children Wasting · 4.6 % (3/65) of under-five children; higher prevalence among females
(Heney et al., 2015)	To evaluate BMI changes among resettled refugee setting	USA	2–18 years	Burundi Somalia Liberia Eritrea DRC Iraq Nepal Bhutan Myanmar	Retrospective Cross-sectional	*N* = 156	On arrivalUnderweight · 5.7 % Overweight · 14.1 % Obesity · 3.2 %
(Meyer et al., 2022)	To compare weight status and body mass index z-scores (BMIz) of refugee children upon arrival	USA	0–18 years	Syria Myanmar Iraq DRC	Comparative cross-sectional	*N* = 139	Underweight · 2.2 % at resettlement and 1.2 % at one year Overweight/obesity · 25.2 % at resettlement and 32.9 % at one year · Refugees had a lower rate of being overweight but increased over time
(Sandell et al., 2017)	To assess the health status and anthropometric changes in resettled refugee children	USA	6 months-18 years	Somalia Ethiopia, Burundi, DRC, Thailand, Myanmar Africa	Retrospective cohort	*N* = 219	Anemia • 33.3 % (72/217) overall; 42.9 % (33/77) among under-five children HBV infection • 3.8 % (80/209) Immune to HBV • 41.8 % (87/208) Parasitic infection • Giardiasis 22.4 % (49/219) and B.hominis, 41.6 % (91/219) Elevated blood lead level • 5.6 % (12/215)
				Myanmar, Bhutan, Iraq, Southeast Asia	Retrospective cohort	*N* = 193	Anemia • 15.4 % (29/186) overall; 23.9 % (11/47) among under-five children Vitamin D deficiency • 87 % (90/104) Immune to HBV • 62.8 % (113/208) Parasitic infection • Giardiasis 12.4 % (24/193) and B.hominis 33.2 % (64/193) EBLL • 7.8 % (12/215)
(Maldari et al., 2019)	To assess the health status of newly arrived Syrian refugees in Australia	Australia	0–18 years	Syria	Retrospective cross-sectional	*N* = 268	Iron deficiency • 38.8 % had low serum iron levels. HBV Vaccination /Immunity to HBV/ • 61.6 % of children were immune to HBV Vitamin B12 Deficiency • 35.1 % (46/131) Vitamin D deficiency • 25.9 % (69/266) Stool parasite (parasitic infection) • Gardia lambia 6.5 % (17/261), Strongyloidiasis 4.1 % (11/266), and Dientamoeba fragilis 8.8 % (23/261)
(Aucoin et al., 2013)	To identify the Vitamin D status of refugees arriving in Canada	Canada	0–18 years	Asia Africa the Middle East South America	Retrospective cross-sectional	*N* = 756	Vitamin D deficiency • 10 % overall; higher among children from the Middle East (25 % (15/59))
(DeVetten et al., 2017)	To assess Parasitic stool testing in newly arrived refugees	Canada	6 months-16 years	Africa Asia the Middle East Europe North America	Retrospective cross-sectional	*N* = 234	Intestinal parasite • 38.5 % (Amoeba and Giardiasis were common parasites); 9.6 % (37/125) among children under five years of age; more prevalent among children from sub-Saharan Africa and Asia.
(Beukeboom and Arya, 2018)	To assess nutritional deficiencies among newly arriving government-assisted refugee children	Canada	≤ 16 years	Iraq Somalia Myanmar Afghanista	Retrospective cross-sectional	*N* = 357	Anemia • 15.7 % (56/356) overall; 25 % among children under-five years of children; higher among children from Somalia Vitamin D deficiency • 25 % (29/116) overall; 30.4 % (7/23) among under-five children; highest in children from the Middle Eastern Regions Vitamin B12 deficiency • 11.2 % (40/357); 3.9 % (3/78) among under-five years of children
(Scott et al., 2015)	To assess hepatitis B status among resettled refugee children	USA	≤ 18 years	Bhutan, Burma, Iraq, and others (Eritrea, Ethiopia, Somalia, and the former Soviet	Retrospective cross-sectional	*N* = 1279	HBV infection • HBV infection (past and current) was reported among 9.5 % of children HBV vaccination (Immunity to HBV) • 51.8 % of children immunized for HBV
(Zwi et al., 2017b)	To assess refugee children's health, development and well‐being over the first year of settlement	Australia	6 months-15 years	Southeast Asia Africa, Eastern Mediterranean	Longitudinal study	*N* = 61	Iron deficiency • 26.2 % Vitamin D deficiency • 16.4 % Chronic disease • 15 % (9.8 % latent TB, 4.9 % schistosomiasis, and chronic HBV 1.6 %)
			≤5 Years				Mild developmental problems • 27 % (4/15) at year two after arrival (four languages domains and two cognitive/ personal-social domains) • 23 % (3/13) under-five children had a mild developmental problem at year three after arrival (two cognitive domains and one fine motor domain)
			4 −15 years				Socioemotional problems • Abnormal total difficulty scores were 12.8 % (5/39) at year two and 10 % (4/39) at year three; the borderline scores were 6 % (2/38) at year two and 3 % (1/38) at year three.
(Salehi et al., 2015)	To assess the health and growth status of immigrant and refugee children	Canada	≤6 years	Middle East Asia Latin America Caribbea Sub-Saharan Africa Southeast Asia Europe	Retrospective cross-sectional	*N* = 145	Anemia • 22.8 % of children were anaemic Iron deficiency • 53.3 % (49/92) HBV infection • 2.5 % (31/122) using hepatitis B surface Antigen. Parasitic infection • 33.6 % (41/122) overall; Giardia lamblia (37.7 %), Dientamoeba histolytica (7.5 %), Dientamoeba fragilis (28.3 %), Ascaris lumbricoides (15.1 %), Enterobius vermicularis (3.8 %), and Hymenolepis (3.8 %) EBLL • 4.9 % (4/81) had lead poisoning
(Rungan et al., 2013)	To assess the health needs of refugee children younger than five years on arrival	New Zealand	≤ 5 years	Middle East Africa Asia America	Retrospective cross-sectional	*N* = 343	Latent TB • 17 %; the rate of latent TB is almost similar across the groups (African 16 %, American 24 %, Asian 24 %, Middle East (ME) 18 %) Iron deficiency • 33 %; the highest rates were in those from the Middle East (50 %), and Africa (42 %) Vitamin D deficiency • 13 % overall; highest in the Middle East group (20 %) and the Asia group (19 %) Intestinal parasite • 11 % overall; the most common parasites were giardia (58 %), followed by Ascaris (16 %), and Trichuris (14 %). Hemoglobinopathies • Reported in 2 % of children Immune to rubella • 50 % (87/174) for children above one year of age; higher in the Americas group (71 %), with 44 % in the Asia group, 34 % in Middle East group, and 14 % in the Africa group Immune to measles • 59 % (117/197) and no difference across countries HBV vaccination status • 68 % (141/207) and most come from countries where the Global Alliance for Vaccines and Immunisation program
(Kloning et al., 2018)	To assess the morbidity profile and sociodemographic characteristics of unaccompanied refugee minors	Germany	10–18 years	Somalia Eritrea Afghanista Syria Unspecified	Retrospective cross-sectional	*N* = 154	Latent TB • 8.2 % (5/61) Bronchitis • 1.9 % (3/154) Scabies • 27.9 % (43/154) Pediculosis • 5.2 % (8/154) Syphilis • 2.4 % (3/126) HBV infection • 8.0 % (9/113) Immune to hepatitis B • 92.8 % (77/83) Hepatitis C virus infection • 1.5 % (1/65) Immunity to hepatitis A • 92.8 % (77/83) Dental caries • 71.4 % (30/42) Parasitic infections • 7.1 % (11/154) overall; Giardia lamblia (5.2 %), Schistosoma mansoni (1.3 %), and Strongyloidiasis stercoralis (0.6 %) Helicobacter pylori • 25.9 % (40/154) had positive using stool antigen test Post-traumatic stress disorder (PTSD) • 25 % (7/28)
(Solberg et al., 2021)	To assess health-related quality of life in refugee minors	Sweden	12–18 years	Afghanista Iraq Syria	Comparative Cros-sectional	*N* = 2559	Health-related quality of life • Refugee children had lower levels of health-related quality of life for psychological well-being and peers and social support
(Yun et al., 2016)	To increase hepatitis B vaccine status among refugee children arriving in USA	USA	0–18 years	Myanmar Somalia Iraq Bhutan Ethiopia Laos	Retrospective cross-sectional	*N* = 937	HBV vaccination • HBV among children born before the national integration of the EPI program was 31.3 % and after the EPI program (69.7 %)
(Gandham et al., 2021)	To determine the prevalence of symptoms of post-traumatic stress in children	Australia	6–11 years	Eastern Mediterranean Southeast Asian African Western Pacific Europe	Retrospective cross-sectional	*N* = 28	PTSD • 42.9 % of children develop PTSD using the child trauma screening questionnaire (CTSQ) six months from arrival (10 checklist)
(Solberg et al., 2020)	To assess nationwide, representative prevalence estimates for symptom-defined PTSD	Sweden	16–18 years	Afghanista Iraq Syria	Cross-sectional	*N* = 1129	PTSD • 42 % of minors develop PTSD • Children from Afghanistan presented the highest prevalence (56.9 %), compared to minors from Iraq (36.8 %) and Syria (33.4 %)
(Anil et al., 2022)	To identify EBLL among refugee children	USA	≤16 years	Afghanista Nepal Africa Malawi Malaysia Thailand Ukraine Syria Jordan Iraq	Retrospective cross-sectional	*N* = 3142	Elevated blood lead level • 18.4 % • 24.5 % (335/1370) for six months to 6 years, of which 145 (43.3 %) were not retested
(Fozouni et al., 2019)	To assess the immunisation status of refugee children	Germany	<18 years	Syria Afghanistan Iraq Moldova	Cross-sectional	*N* = 150	Full immunization • 59.3 % of parents reported their children were fully immunised • 51.2 % (40/78) among children under five years of age
(Raymond et al., 2013)	To assess Elevated Blood Lead Levels among refugee children	USA	<16 years	Not specified	Longitudinal study	*N* = 1007	EBLL • 2.5 % had EBLL within three months of arrival
(Pezzi et al., 2019)	To assess BLL among resettled refugee children	USA	Six months-16 years	Nepal Thailand Iraq Kenya India Afghanista Ethiopia Jordan Syria Uganda Malaysia	Cross sectional	*N* = 27,284	EBLL • 19.3 % (5275), of which 22.8 % (2836) were under seven years of age. Anemia • 3.7 % (327/8951) Wasting • 4.7 % (423) Stunting • 20.8 % (1865)
(Geltman et al., 2019)	To assess the trends of ELBL	USA	<7 years	African Europe Eurasia Asia Western hemisphere	Comparative cross-sectional	*N* = 3054	EBLL • 49.8 %, had EBLL Anemia • 26.0 % (670) Latent TB • 9.7 % (296) Intestinal parasite (IP) • 19.9 % (363) Stunting • 9.8 % (299) Wasting • 8.3 % (252) Underweight • 10.1 % (308)
(Shakya and Bhatta, 2019)	To assess the prevalence of ELBL and demographic characteristics	USA	0–18 years	South Asia Africa Ukraine	Retrospective cross-sectional study	*N* = 5661	EBLL • 22.3 % overall; 27.1 % (643/2378) among children under six years of age; highest among children from Afghanistan and Bhutan, 75.7 %
(Lupone et al., 2020)	To assess lead exposure among refugee children	USA	0–16 years	Africa Middle East Southeast Asia Eastern Europe	Retrospective cross-sectional study	*N* = 705	EBLL • 17 % of children had ELBL. • 10 % had ELBL upon follow-up; 8.3 % were new exposures Anemia • 16.0 % (113) of children had anemia. • Increased risk of increased blood lead levels and coexistence with children under 5 years.
(Pezzi et al., 2022)	To assess blood lead levels among resettled refugee children	USA	0–16 years	Afghanista	Retrospective cross-sectional	*N* = 4130	EBLL • 32 % overall; 47 % (526/1120) among children less than two years of age Anemia • 5.1 % (212) Stunting • 8.8 % (365)
(Seifu et al., 2020)	To evaluate the status of blood lead levels among refugee children on arrival	USA	6 months- 16 years	Afghanista Nepal Iraq Myanmar The Republic of Congo Thailand Russia Colombia Kenya Somalia Ethiopia Syria Tanzania Burundi Lebanon Liberia Zaire	Retrospective cross-sectional	*N* = 301	EBLL • 13 % (24/63) overall; higher among children from Afghanistan
(Kotey et al., 2018)	To examine blood lead levels in post-resettlement	USA	≤15 years	Asia Latin America the Middle East Sub-Sharan Africa	Retrospective cross-sectional	*N* = 1722	EBLL • 11.2 % overall; 14.3 % (45/314) among children ≤3 years of age
(Pavlopoulou et al., 2017)	To evaluate clinical and laboratory characteristics of immigrants and refugee children on arrival	Greece	1–14 years	Asia Africa Europe	Retrospective cross-sectional	*N* = 162	Anemia • 12.3 % Low ferritin level • 12.3 % Immune to HBV • 51.2 % BCG vaccination • 90.7 % Dental caries • 24.7 % EBLL • 23.7 % (27/114) overall; more prevalent among under-five children, and children from Asia Latent TB • 4.9 % (8) had latent TB using mantoux test
(Buchmüller et al., 2018)	To explore mental health status and syndrome among refugee children	Germany	1.5–5 years	Syria Iraq	Cross-sectional	*N* = 35	Mental health problems • Increased level of anxiety/depression/ attention problems • Increased withdrawal behavior and syndrome scales of internalising difficulties reported
(Riatto et al., 2018)	To assess the oral health status of Syrian refugee children	Spain	5–13 years	Syria	Cross-sectional	*N* = 156	Dental caries • Dental caries (70.5 %), permanent dentition (27.6 %), and deciduous dentition (55.8 %); higher among younger children (<11 years) (74 %) than children between 11–13 years of age (59 %)
(Calderon and Rominger, 2019)	To assess the health profile of refugee children	USA	0–18 years	Syria Iraq Jordan Turkey Egypt Lebanon	Retrospective cross-sectional	*N* = 262	Dental caries • 60.6 % (146/241) Underweight • 70.2 % Anemia • 19.2 % (31/161) EBLL • 1.2 % (3/240) Latent TB • 20 % (9/45) Haematuria • 34.3 % (79/230) Proteinuria • 17.4 % (40/230) Imprisonment, torture, or violence • 21.7 % (53/244) Vision changes • 28.1 % (50/178)
(Theuring et al., 2016)	To assess infectious disease among unaccompanied refugee minors	Germany	under 18 years	Syria Sub-Saharan Africa Afghanista	Cross-sectional study	*N* = 1248	Intestinal infection · 19.6 % Contagious diseases • 15.3 % (Giardia duodenales, hepatitis B, scabies, cutaneous fungal infection, unspecified diarrhea, syphilis)
(Macfarlane et al., 2023)	To assess tuberculosis among refugees from Afghanistan	United Kingdom	0–16 years	Afghanista	Cross-sectional study	*N* = 347	Latent tuberculosis · 1.5 % (47/311)
(Zwi et al., 2017a)	To screen the health of newly arrived children	Australia	0–15 years	Africa Southeast Asia Eastern Mediterranean	Cross-sectional study	*N* = 367	Anemia • 10.9 % (34/312) Iron deficiency • 70.5 % (208/295) Vitamin deficiency • 26.8 % (83/310) Schistosomiasis • 14.6 % (31/212) Malaria • 4.0 % (12/316) HBV • 4.1 % (11/265) Strongyloidiasis • 2.9 % (6/208)
(Mockenhaupt et al., 2016)	To assess illness among unaccompanied minors	Germany	under 18 years	Syrian	Cross-sectional study	*N* = 488	Intestinal parasite • 22 % diagnosed with at least one intestinal parasite (E.g., Giardia duodenalis (7 %), Blastocystis sp. (12 %), and other non-pathogenic protozoa (6 %)) • 7 (1.4 %) tested positive for schistosomiasis
(Heudorf et al., 2016)	To assess multidrug resistance bacteria in unaccompanied refugee	Germany	under 18 years	Africa Afghanista Iraq Pakistan	Cross-sectional study	*N* = 119	Multidrug resistant Enterobacteriaceae • Extended spectrum beta lactamase (ESBL) was detected in 35 %, with 8 % of 3-multidrug-resistant Gram-negative bacteria (fluoroquinolones)
(Janda et al., 2020)	To assess comprehensive infectious disease among refugee children	Germany	under 18 years	Africa Afghanista Syria	Cross-sectional study	*N* = 890	Scabies • 14.2 % Dental caries • 65.1 % HBV infection • 7.7 % (60/776) Intestinal parasite • 16 % (19/119); intestinal schistosomiasis (6.7 %), Giardiasis (5 %). Latent TB • 30.9 % (38/123) Pulmonary TB • 1.7 % (15/874) HIV infection · 0.4 % (3/760)
(Jensen et al., 2014)	To examine the development of mental health problems among unaccompanied refugee minors	Norway	12.5–18 years	Afghanista Eritrea Somalia Sri Lanka	Longitudinal study	*N* = 75	Mental health problems • Level of PTSD, depression, anxiety, or externalising problems increased shortly after arrival to nearly two years later • An increase in the number of reported stressful life events
(Hanes et al., 2017)	To assess adversity and resilience among resettling paediatric refugees	Australia	2–16 years	Southeast Middle East Africa	Cohort	*N* = 204	Mental health problems • 37.3 % had at least one psychological symptom (sleep disturbance, separation, anxiety, excessive crying, secondary enuresis, aggression, nightmares) • Peer problem scores were consistently elevated

### 2.6 Critical quality appraisal

We assessed the quality of the included studies using JBI quality assessment criteria. The appraisal tool consists of nine items for cross-sectional studies and 11items for cohort/longitudinal studies (Barker et al., 2023). We used percentage-based scoring to classify studies as high, medium, or low quality. Studies with ≥ 80 % of applicable JBI criteria classified as high quality, 50–79 % criteria as medium quality, and ≤50 % as low quality.

## 3 Results

### 3.1 Search results

**Fig. 1:**
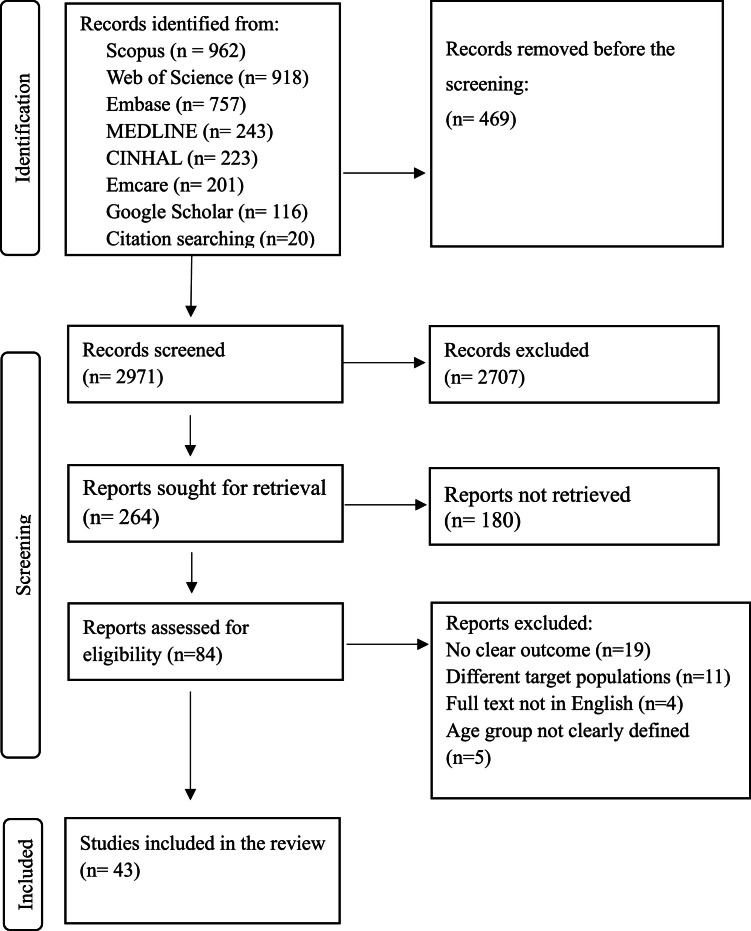
PRISMA flow diagram.

We retrieved a total of 3440 articles. Following the removal of duplicates, 2971 articles were screened based on their titles and abstracts, of which 84 articles were selected for full-text review. Of these, 43 studies met the inclusion criteria and were included in the scoping review (Fig. 1).

### 3.2 Characteristics of the included studies

A total of 43 studies were included in this scoping review. Of the 43 studies, 17 were conducted in the USA (Anil et al., 2022; Calderon and Rominger, 2019; Dawson-Hahn et al., 2016; Geltman et al., 2019; Heney et al., 2015; Kotey et al., 2018; Lupone et al., 2020; Meyer et al., 2022; Pezzi et al., 2022, 2019; Raymond et al., 2013; Sandell et al., 2017; Scott et al., 2015; Seifu et al., 2020; Shah et al., 2014; Shakya and Bhatta, 2019; Yun et al., 2016), seven in Germany (Buchmüller et al., 2018; Fozouni et al., 2019; Heudorf et al., 2016; Janda et al., 2020; Kloning et al., 2018; Mockenhaupt et al., 2016; Theuring et al., 2016), six in Australia (Gandham et al., 2021; Hanes et al., 2017; Maldari et al., 2019; Newman et al., 2019; Zwi et al., 2017b), four in Canada (Aucoin et al., 2013; Beukeboom and Arya, 2018; DeVetten et al., 2017; Salehi et al., 2015), three in Greece (Grammatikopoulou et al., 2019; Pavlopoulou et al., 2017; Walpole et al., 2018), two in Sweden (Solberg et al., 2021), one in New Zealand (Rungan et al., 2013), UK (Macfarlane et al., 2023), Norway (Jensen et al., 2014), and Spain (Riatto et al., 2018).

Majority, 38 studies are cross-sectional (Anil et al., 2022; Aucoin et al., 2013; Beukeboom and Arya, 2018; Buchmüller et al., 2018; Calderon and Rominger, 2019; Dawson-Hahn et al., 2016; DeVetten et al., 2017; Fozouni et al., 2019; Gandham et al., 2021; Geltman et al., 2019; Grammatikopoulou et al., 2019; Heney et al., 2015; Heudorf et al., 2016; Janda et al., 2020; Kloning et al., 2018; Kotey et al., 2018; Lupone et al., 2020; Macfarlane et al., 2023; Maldari et al., 2019; Meyer et al., 2022; Mockenhaupt et al., 2016; Newman et al., 2019; Pavlopoulou et al., 2017; Pezzi et al., 2022, 2019; Riatto et al., 2018; Rungan et al., 2013; Salehi et al., 2015; Scott et al., 2015; Seifu et al., 2020; Shah et al., 2014; Shakya and Bhatta, 2019; Solberg et al., 2021; Theuring et al., 2016; Walpole et al., 2018; Yun et al., 2016; Zwi et al., 2017b), three longitudinal studies (Jensen et al., 2014; Raymond et al., 2013; Zwi et al., 2017b), and two cohort studies (Hanes et al., 2017; Sandell et al., 2017). Most refugee children in these studies originated from Southeast Asia, Africa, and the Middle East.

The sample size ranged from 35 participants (Buchmüller et al., 2018) to 27,284 participants (Pezzi et al., 2019). The study population ranged from 6 months to 18 years, with most studies (39) focused on children under 18 years old, with only three studies specifically focused on children under five years old (Buchmüller et al., 2018; Rungan et al., 2013; Walpole et al., 2018), and one study among under seven years old children (Geltman et al., 2019) (Table 1).

**Fig. 2:**
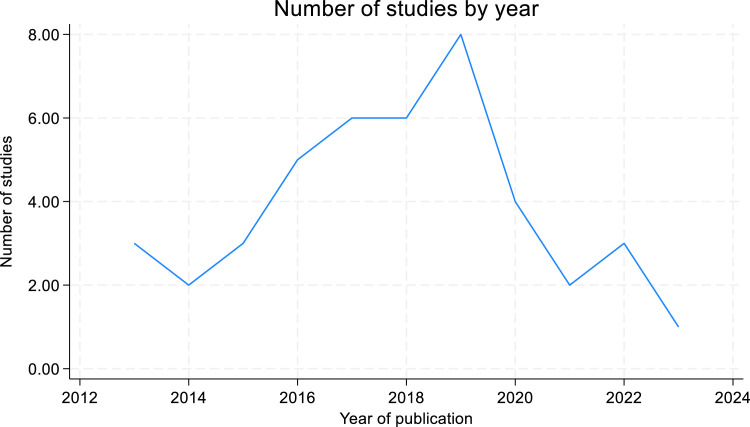
Trends of publication of the studies included in ten-year periods.

The included studies were published between 2013 and 2023, with an increase in publications after 2016 and decrease after 2020. Most (29) studies were published between 2016 and 2020, and the publication trend increased significantly in 2019 (8) (Fig. 2).

Geographical variations in health outcomes of refugee children

The scoping review revealed that the burden of health indicators varied across countries. Nearly half, 20 studies (Anil et al., 2022; Beukeboom and Arya, 2018; Calderon and Rominger, 2019; Dawson-Hahn et al., 2016; Geltman et al., 2019; Janda et al., 2020; Kloning et al., 2018; Lupone et al., 2020; Maldari et al., 2019; Newman et al., 2019; Pavlopoulou et al., 2017; Pezzi et al., 2022, 2019; Rungan et al., 2013; Salehi et al., 2015; Sandell et al., 2017; Shah et al., 2014; Theuring et al., 2016; Zwi et al., 2017a, 2017b) reported diverse but overlapping child health outcomes, which varied by country.

**Fig. 3:**
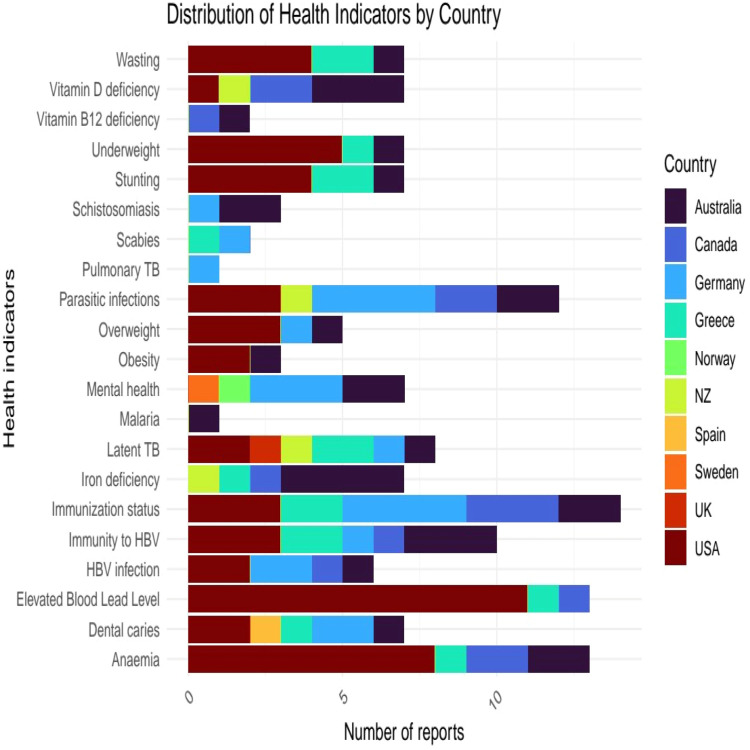
Distribution of child health indicators of refugees upon resettlement in high-income countries.

As shown in the stacked bar graph below, studies from Australia and the USA showed the highest diversity of health problems. Studies from the USA often reported elevated blood lead levels (1), anemia (8), underweight (5), obesity (3), and overweight (2). Immunization status (e.g., immunity to HBV, vitamin A, Bacille Calmette Guerin (BCG), measles, and rubella) was reported in five countries; majority of reports were related to immunity to HBV. Micronutrient deficiencies (e.g., iron and vitamin D) were commonly reported in studies conducted in Australia and Canada. Other health problems reported in specific regions include schistosomiasis (two reports in Australia and one report in Germany), active tuberculosis (one report in Germany), BCG vaccination status (one in Greece), and malaria (one report in Australia) (Fig. 3).

### 3.3 Thematic synthesis of studies

The thematic synthesis of the health of refugee children upon resettlement in high-income countries revealed a wide range of health problems. We categorized the findings into seven key health outcome themes, such as nutritional status, infectious diseases, parasitic infection, blood lead level, oral health, immunization status and mental health. Each theme has various outcome measures as shown in Table 2.

**Table 2:** Summary of health indicators among refugee children upon resettlement in high-income countries.

Health status (theme)	Health indicators/measures	Findings (% range)	Source
Nutritional status and growth	Underweight	2.2 to 70.2	(Calderon and Rominger, 2019; Geltman et al., 2019; Grammatikopoulou et al., 2019; Heney et al., 2015; Meyer et al., 2022; Newman et al., 2019; Shah et al., 2014)
	Stunting	7.3 to 20.8	(Dawson-Hahn et al., 2016; Geltman et al., 2019; Grammatikopoulou et al., 2019; Newman et al., 2019; Pezzi et al., 2022, 2019; Shah et al., 2014; Walpole et al., 2018)
	Wasting	2.3 to 17.3	(Dawson-Hahn et al., 2016; Geltman et al., 2019; Grammatikopoulou et al., 2019; Newman et al., 2019; Pezzi et al., 2019; Shah et al., 2014)
	Overweight	7.6 to 25.2	(Dawson-Hahn et al., 2016; Heney et al., 2015; Meyer et al., 2022; Newman et al., 2019)
	Obesity	3.2 to 5.9	(Dawson-Hahn et al., 2016; Heney et al., 2015; Meyer et al., 2022)
	Vitamin B12 deficiency	35.1 to 11.2	(Beukeboom and Arya, 2018; Maldari et al., 2019)
	Vitamin D deficiency	10 to 87	(Aucoin et al., 2013; Beukeboom and Arya, 2018; Maldari et al., 2019; Newman et al., 2019; Rungan et al., 2013; Sandell et al., 2017; Zwi et al., 2017b)
	Iron deficiency	12.3 to 53.3	(Maldari et al., 2019; Newman et al., 2019; Pavlopoulou et al., 2017; Rungan et al., 2013; Salehi et al., 2015; Zwi et al., 2017a, 2017b)
	Anaemia*	5.1 to 33.3	(Beukeboom and Arya, 2018; Calderon and Rominger, 2019; Geltman et al., 2019; Lupone et al., 2020; Newman et al., 2019; Pavlopoulou et al., 2017; Pezzi et al., 2022; Sandell et al., 2017; Shah et al., 2014) The highest in African and Southeast Asian refugees and common among under five children (Newman et al., 2019)
Infectious diseases	Hepatitis B virus (HBV)	1.6 to 9.5	(Janda et al., 2020; Salehi et al., 2015; Sandell et al., 2017; Scott et al., 2015; Theuring et al., 2016; Yun et al., 2016; Zwi et al., 2017a, 2017b)
	Latent TB	4.9 to 30.9	(Calderon and Rominger, 2019; Geltman et al., 2019; Janda et al., 2020; Kloning et al., 2018; Macfarlane et al., 2023; Pavlopoulou et al., 2017; Rungan et al., 2013; Zwi et al., 2017b)
	Active TB	1.7	(Janda et al., 2020)
Parasitic infection	Stool parasite	7.1 to 41.6	(Geltman et al., 2019; Kloning et al., 2018; Maldari et al., 2019; Mockenhaupt et al., 2016; Rungan et al., 2013; Salehi et al., 2015; Sandell et al., 2017; Shah et al., 2014; Theuring et al., 2016)
Neglected tropical diseases	Scabies	14.2 to 27.9	(Janda et al., 2020; Kloning et al., 2018; Mockenhaupt et al., 2016; Theuring et al., 2016; Zwi et al., 2017a, 2017b)
	Schistosomiasis	4.9 to 14.6	
Oral health	Dental caries	24.7 to 71.4	(Calderon and Rominger, 2019; Janda et al., 2020; Kloning et al., 2018; Newman et al., 2019; Pavlopoulou et al., 2017; Riatto et al., 2018; Shah et al., 2014) More prevalent among refugees from Thailand and Malaysia (Shah et al., 2014),
Blood lead level	EBLL	2.5 to 49.8	(Anil et al., 2022; Geltman et al., 2019; Kotey et al., 2018; Lupone et al., 2020; Pavlopoulou et al., 2017; Pezzi et al., 2022, 2019; Raymond et al., 2013; Salehi et al., 2015; Sandell et al., 2017; Seifu et al., 2020; Shakya and Bhatta, 2019) Increase among children less than five years (Pavlopoulou et al., 2017) and highest among children from Afghanistan and Bhutan than children from Africa (Shakya and Bhatta, 2019)
Immunization status	Immune to hepatitis B	31.3 to 92.8	(Kloning et al., 2018; Maldari et al., 2019; Pavlopoulou et al., 2017; Rungan et al., 2013; Scott et al., 2015)
	Immune to rubella	50	(Rungan et al., 2013)
	Immune to measles	59	
	BCG vaccination	90.7	(Pavlopoulou et al., 2017)
Mental health problems	PTSD	25 to 42.9	(Gandham et al., 2021; Hanes et al., 2017; Jensen et al., 2014; Kloning et al., 2018; Solberg et al., 2020) Increased level of anxiety/depression/ attention problems (Buchmüller et al., 2018)

#### 3.3.1 Nutritional status and growth

Findings revealed that refugee children often settled in high-income countries with a higher burden of malnutrition, including acute and chronic malnutrition. Of the included studies, 20 studies reported on nutritional status (Aucoin et al., 2013; Beukeboom and Arya, 2018; Calderon and Rominger, 2019; Dawson-Hahn et al., 2016; Geltman et al., 2019; Grammatikopoulou et al., 2019; Heney et al., 2015; Lupone et al., 2020; Maldari et al., 2019; Meyer et al., 2022; Newman et al., 2019; Pavlopoulou et al., 2017; Pezzi et al., 2022; Rungan et al., 2013; Salehi et al., 2015; Sandell et al., 2017; Shah et al., 2014; Walpole et al., 2018; Zwi et al., 2017a, 2017b), with varied nutritional deficiencies such as underweight (2.2 to 70.2 %) (Calderon and Rominger, 2019; Geltman et al., 2019; Grammatikopoulou et al., 2019; Heney et al., 2015; Meyer et al., 2022; Newman et al., 2019; Shah et al., 2014), overweight (7.6 to 25.2 %) (Dawson-Hahn et al., 2016; Heney et al., 2015; Meyer et al., 2022; Newman et al., 2019), obesity (3.2 to 5.9 %) (Dawson-Hahn et al., 2016; Heney et al., 2015; Meyer et al., 2022), and vitamin D deficiency (10 to 87 %) (Aucoin et al., 2013; Beukeboom and Arya, 2018; Maldari et al., 2019; Newman et al., 2019; Rungan et al., 2013; Sandell et al., 2017; Zwi et al., 2017b), (Table 2).

Anemia represents a substantial health issue among the refugee children characterized by multifactorial aetiologies, demonstrating a prevalence ranging from 5.1 % to 33.3 % (Beukeboom and Arya, 2018; Calderon and Rominger, 2019; Geltman et al., 2019; Newman et al., 2019; Pavlopoulou et al., 2017; Pezzi et al., 2022, 2019; Salehi et al., 2015; Sandell et al., 2017; Shah et al., 2014; Zwi et al., 2017a) and disproportionately affect children under-five years and refugees from Africa (Shah et al., 2014).

While most studies did not stratify the burden of health problems based on refugees’ country of origin, evidence from Canada and New Zealand revealed that vitamin D deficiency was more prevalent among refugees from the Middle East and South Asia than among refugees from other regions (Aucoin et al., 2013; Rungan et al., 2013).

#### 3.3.2 Infectious diseases

Infectious diseases such as latent TB and hepatitis B (Calderon and Rominger, 2019; Geltman et al., 2019; Janda et al., 2020; Kloning et al., 2018; Macfarlane et al., 2023; Pavlopoulou et al., 2017; Rungan et al., 2013; Salehi et al., 2015; Sandell et al., 2017; Theuring et al., 2016; Zwi et al., 2017a, 2017b) %) were reported. Malaria (Zwi et al., 2017a), active TB, and HIV (Janda et al., 2020) were less reported.

#### 3.3.3 Parasitic infections

Stool parasites (DeVetten et al., 2017; Geltman et al., 2019; Janda et al., 2020; Kloning et al., 2018; Maldari et al., 2019; Mockenhaupt et al., 2016; Rungan et al., 2013; Salehi et al., 2015; Sandell et al., 2017; Shah et al., 2014; Theuring et al., 2016; Zwi et al., 2017a), including Giardia lamblia, Strongyloidiasis, and Di entamoeba fragilis, have been frequently reported, with a higher prevalence among refugees from Africa (DeVetten et al., 2017).

#### 3.3.4 Neglected tropical diseases

Findings revealed that scabies and schistosomiasis were reported among refugee children with a prevalence range of 14.2–27.9 % and 4.9 to 14.9 % respectively (Kloning et al., 2018; Theuring et al., 2016; Zwi et al., 2017a, 2017b).

#### 3.3.5 Oral health

Dental caries were prevalent among refugee children, with the prevalence range of 24.7 to 71.8 % (Calderon and Rominger, 2019; Kloning et al., 2018; Newman et al., 2019; Pavlopoulou et al., 2017; Riatto et al., 2018; Shah et al., 2014) and a higher burden among refugees from Thailand and Malaysia compared to refugees from Africa and Bhutan (Shah et al., 2014)

#### 3.3.6 Elevated blood lead level

Elevated blood lead levels have been among the most frequently mentioned in the included studies. Of these, majorities were conducted in the USA and the burden increased among children under five years (Anil et al., 2022; Calderon and Rominger, 2019; Geltman et al., 2019; Kotey et al., 2018; Lupone et al., 2020; Pavlopoulou et al., 2017; Pezzi et al., 2022, 2019; Raymond et al., 2013; Salehi et al., 2015; Sandell et al., 2017; Seifu et al., 2020; Shakya and Bhatta, 2019). One study from the USA (Lupone et al., 2020) found that 8.3 % of new exposures occurred after resettlement.

#### 3.3.7 Immunization status

Immunization status among refugee children was reported, with the majority on immunity to immunity to the hepatitis B virus, 31.3 to 92.8 % (Fozouni et al., 2019; Kloning et al., 2018; Maldari et al., 2019; Pavlopoulou et al., 2017; Rungan et al., 2013; Sandell et al., 2017; Scott et al., 2015; Yun et al., 2016) and only a single report on immunity for measles (50 %), rubella (59 %) (Rungan et al., 2013) and BCG vaccination (90.7 %) (Pavlopoulou et al., 2017).

#### 3.3.8 Mental health

Posttraumatic stress disorder (PTSD) has been a prevalent mental health problem affecting 25 to 42.9 % of refugees in the included studies and has increased among minors. In addition, children experienced psychological problems (e.g., anxiety, depression, excessive crying), behavioral disturbances (e.g., secondary enuresis, aggression), sleep disturbances (e.g., nightmares, insomnia), social disturbances (e.g., withdrawal behavior, problems with peers), and increased vulnerability to stressful life events (Buchmüller et al., 2018; Gandham et al., 2021; Hanes et al., 2017; Jensen et al., 2014; Kloning et al., 2018; Solberg et al., 2020).

### 3.4 Quality assessment of included studies

We conducted a critical quality appraisal of 43 studies (38 cross-sectional, three longitudinal, and two cohort studies) using the JBI checklist (Barker et al., 2023). Most of the included studies had a clear study design and report. Based on these criteria, 89.5 % of cross-sectional studies and 40 % of cohort studies (Jensen et al., 2014; Raymond et al., 2013), classified as high quality overall (≥ 80 % criteria met). However, some methodological inconsistencies had been identified. For instance, 13.2 % of cross-sectional studies (Meyer et al., 2022; Seifu et al., 2020) failed to address confounding factors. Additionally, one study (Buchmüller et al., 2018) identified statistical reporting and measurement of exposure variables (not clear), which were classified as medium or low quality evidence. Three studies (Hanes et al., 2017; Sandell et al., 2017; Zwi et al., 2017b) were limited by a lack of strategies to handle incomplete follow-up, revealing medium to low quality evidence. (Annex 3)

## 4 Discussion

This scoping review provides evidence on the health of refugee children upon arrival in high-income countries, highlighting challenges related to malnutrition, infectious diseases, neglected tropical diseases, immunization status, environmental health risks (lead poisoning), dental caries, and mental health problems. While high-income countries have robust healthcare systems, refugee children arrive with various and interconnected health problems that require tailored interventions to address their specific needs.

### 4.1 Nutritional status

One of the significant health problem reported in the review was high burden of malnutrition, including stunting, wasting, underweight, obesity, overweight, and vitamin deficiencies (e.g., vitamin D, vitamin B12) (Aucoin et al., 2013; Beukeboom and Arya, 2018; Maldari et al., 2019; Newman et al., 2019; Zwi et al., 2017a, 2017b) The burden of malnutrition varies across countries and population groups (e.g., age and country of origin). For instance, vitamin D deficiency has been reported with a higher prevalence among refugees from South Asia and the Middle East (Newman et al., 2019), highlighting the need to identify risk factors associated with nutritional supplementation, dietary intake, sunlight exposure practices, access to services, economic instability, and broader socioecological issues (Bendavid et al., 2021). Anemia is also one of the health problems reported to be more prevalent among refugees from Africa and Southeast Asia (Newman et al., 2019) and under five children, 33.3 % (Sandell et al., 2017) which is lower than the global burden of 44.0 % among children aged 6–59 months (Kay et al., 2019), and Africa, 59 % (Tadesse et al., 2022). This disparity could be due to differences in the study population groups, sample sizes, study contexts, and data collection methods. These variations highlight the need for targeted health interventions and support systems for refugee children's nutritional needs. Addressing these nutritional needs is crucial for immediate benefits and for preventing long-term health issues, like noncommunicable diseases (Kirolos et al., 2024). Future research should investigate the contributing factors of malnutrition in refugee populations and their long-term health effects, as the double burden (obesity and overweight) typically rises 2–3 years after resettlement (Heney et al., 2015).

### 4.2 Infectious diseases

The findings revealed that refugee children presented with infectious diseases such as hepatitis, latent tuberculosis, active tuberculosis, and various parasitic infections (Janda et al., 2020; Zwi et al., 2017a). This is supported by a review, which found high prevalence of HIV and active tuberculosis among African refugees, and LTB, hepatitis B, and hepatitis C among Asian and Eastern Mediterranean refugees (Eiset and Wejse, 2017; Taha et al., 2023). However, UNHCR report indicated that malaria and respiratory infections are significant causes of morbidity and mortality in refugee settings, especially among African and Asian children (UNHCR, 2023c). The high burden of infectious diseases among the refugee population may be due to factors across migration journey and refugees who were from countries with a high burden of infectious diseases. For instance, children from African countries are at higher risk of presenting with tuberculosis, while those from Asian regions are more likely to exhibit hepatitis B virus (HBV) infection. Factors such as disrupted healthcare systems, inadequate healthcare coverage, malnutrition, overcrowded living conditions, insufficient vaccination coverage, and ongoing conflict (Altare et al., 2019; Kadir et al., 2019) exacerbate their vulnerability to infectious diseases. Moreover, delayed access to healthcare, discrimination, limited cultural competence among healthcare providers, acculturative stress, and insufficient health screening mechanisms also contribute to the persistence of health problems (Cayreyre et al., 2024; WHO, 2021). The findings suggest the importance of addressing infectious diseases among refugee children, who possess diverse migration experiences, originate from a wide range of geographical regions, each with a distinct epidemiological profile, and are resettled in different host countries with varying health care systems.

### 4.3 Neglected tropical diseases

Neglected Tropical Diseases (NTDs) constitute significant yet underrecognized health challenges among refugee children, especially those originating from or migrating through NTD-endemic regions. The present review has identified the presence of schistosomiasis and scabies within the refugee population (Kloning et al., 2018; Theuring et al., 2016; Zwi et al., 2017a, 2017b). The burden of scabies may be related to refugee children are more likely to be subjected to overcrowded conditions, inadequate hygiene facilities, and insufficient health care access. Furthermore, the occurrence of schistosomiasis at 14.6 % is lower compared to the prevalence rates documented in Germany, 18.4 % (Asundi et al., 2019). The observed discrepancies may be attributed to differences in the demographic composition of the study populations and the methodology employed. The German study encompassed both adolescents and immigrants. In contrast, the current study relied on a limited sample size and utilized stool samples (Zwi et al., 2017a), which may lead to a potential underdiagnosis (Kinkel et al., 2012). This variation in methodology may need conducting larger and more representative studies that employ standardized data collection methods, as such studies are essential for accurately estimating the epidemiology within the refugee population and for devising targeted interventions aimed at eradicating schistosomiasis in all affected regions by the year 2030 (WHO, 2022a). The findings related to schistosomiasis might suggest a lack of premigration screening processes and limited treatment options. The comprehensive screening for NTDs within the refugee population is crucial, as these diseases are linked to chronic morbidity, including conditions such as anaemia, stunted growth, impaired neurodevelopment, diminished quality of life, and ongoing child health issues (Abdulrahman, 2016; Khuri et al., 2022).

### 4.4 Immunization status

The current review findings mainly reported on immunity to the hepatitis B virus and reported less on immunization status (Fozouni et al., 2019; Kloning et al., 2018). This may be related to the fact that refugees often present with incomplete vaccination documentation and/or insufficient evidence of immunity against rubella, measles, hepatitis A, and Bacillus Calmette-Guérin (BCG), among other vaccine-preventable conditions. This is further attributed to the disruption of healthcare systems in the refugees' countries of origin, restricted access to healthcare services, a lack of trust in the healthcare system, constrained access to intermediary countries, insufficient information regarding vaccination, and challenges related to resources (Daniels et al., 2022; Mipatrini et al., 2017). Variations in immunization policies across countries may result in disparate access to and uptake of vaccines among refugee children (Elharake et al., 2022). For instance, immunity to rubella is particularly low among refugees originating from Africa, the Middle East, and Asia (Rungan et al., 2013). To effectively meet the specific immunization needs of refugee children from diverse backgrounds and migration experiences, it is essential to adopt standardised, evidence-based screening and targeted immunization strategies.

### 4.5 Blood lead level

Our review findings demonstrated a significant burden of disparities in elevated blood lead levels, with a higher burden among children under five years of age (Calderon and Rominger, 2019; Geltman et al., 2019; Pavlopoulou et al., 2017; Pezzi et al., 2019; Raymond et al., 2013; Shah et al., 2014). This may be related to differences in lead exposure risk that vary across countries of origin, socioeconomic status, fetomaternal transmission, housing conditions, geographical location, public health interventions, and the resettlement environment. Refugee children are especially susceptible to lead exposure from multiple sources, including industrial emissions, aging residential structures, leaded gasoline, lead-based paint materials, indoor dust, and polyvinyl chloride toys (Kordas et al., 2018; Njati and Maguta, 2019; Obeng-Gyasi, 2019). This is supported by the findings from the United States, which identified new diagnoses of lead exposure during follow-up assessments (Shakya and Bhatta, 2019), suggesting a new lead exposure or deficiencies in intervention measures.

The implications of lead exposure on child health are substantial, impacting various body systems, including neurological disorders, renal impairment, cognitive deterioration, behavioral disturbance, attention deficits, and developmental growth concerns (Miracle, 2017), further exacerbated among children with dietary deficiencies in the essential nutrients calcium, iron, and vitamin C (Zhang et al., 2013). This effect may have contributed to neurological disorders, which are predominant contributors to morbidity and disability on a global scale (WHO, 2024a). Such empirical evidence highlights the need for source identification, targeted interventions, and control of the production of lead-containing products, such as lead-based paint, as endorsed by the WHO (WHO, 2023d). This aligns with Sustainable Development Goal 3, target 3.9, aimed at reducing morbidity and mortality related to hazardous environmental exposures (Clark et al., 2020) could further guide strategies to improve refugee child health outcomes

The burden of health status reporting varies significantly based on the country of origin, timing of resettlement, data collection methods, and health policies of the refugee-hosting country (Hvass and Wejse, 2017; WHO, 2022). Despite efforts to research the health status of refugee children, significant gaps remain in understanding their vulnerability and the impact of their migration experiences. Migration leads to violence, kidnapping, detention, and human trafficking, creating additional health and well-being challenges in host countries (Aghajafari et al., 2020; WHO, 2021a; Xu and Saito, 2023). However, the availability of data on the burden of disability and violence experienced by refugee children during their resettlement is limited (Crock et al., 2017), highlighting the need for refugee reception countries to prioritize the health of refugees and implement comprehensive health programs (Blitz et al., 2017; Willey et al., 2018; Yelland et al., 2015).

### 4.6 Strengths and limitations of the study

We used a comprehensive search strategy across multiple databases to capture a range of refugee child health outcomes across numerous high-income countries, thereby broadening the mapping of available evidence and identifying gaps. We also extract data specifically for the target populations (refugee children under 18 years), as some studies were conducted including other migrants and asylum seekers. This review identifies key health problems refugee children faced during resettlement phases in ten high-income countries, reveals significant research gaps, and sets priorities for future studies. The review identified inconsistencies in the literature and methods, especially in controlling confounding factors and maintaining follow-up, emphasizing the need for standardized methodologies. In addition, the article search, which was restricted to English-language publications and specific years, may have overlooked relevant studies published in other languages.

## 5 Conclusions and implications of the study

Studies from ten high-income countries identify children resettled with a multitude of health issues, such as malnutrition, neglected tropical diseases, poor immunization status, infectious diseases, lead poisoning, oral health problems, and mental health problems. Comprehensive screening, intersectoral collaboration, and targeted interventions are essential to promote refugee child health, particularly during the early stages of life. Standardized post-resettlement health reporting are crucial for refugee children resettled in high-income countries to gather comparable data and facilitate cross-country comparisons and evidence-based policymaking. Post resettlement health assessments screening should be done for infectious diseases, nutritional status, environmental exposures, oral health, endemic diseases, and mental health, aligning with the WHO plan for refugee and migrant health (WHO, 2024b). Refugee host countries need to identify and prioritize child health problems to advocate for enhanced health policies for resettled refugee population. Subsequent research should investigate health disparities across the life course (birth, neonatal, under five and later childhood) and compare with other population groups. Future research should also focus on longitudinal studies to better address health trajectories over time and across generations.

## Abbreviation/acronyms

Cumulative Index to Nursing and Allied Health Literature: CINHAL; Excerpta Medica Database: EMBASE; Elevated Blood Lead Level: EBLL; Joanna Briggs Institute: JBI; Neglected Tropical Diseases: NTDs; Post Traumatic Stress Disorder: PTSD; Systematic Reviews and Meta-analysis extension for Scoping Review: PRISMA-ScR; United Nations High Commissioner for Refugees: UNHCR; United Nations Children's Fund: UNICEF; World Health Organization: WHO.

## Ethical approval and consent to participate

Not applicable.

## Consent to publication

Not applicable.

## Funding

This review was supported by the 10.13039/501100000925National Health and Medical Research Council New Ideas Grant (grant number: 2011883), "Creating Health in a New Home: A Transformative Approach to Building for Refugee Health Across Generations." The funder did not have a role in the design, literature search, synthesis, or writing.

## Availability of data and materials

All relevant data are within the manuscript.

## Authors’ Contributions

Binyam Birhane (BB), Angela Dawson (AD), and Andrew Hayen (AH) formulated the research question. BB performed a comprehensive literature search, led the screening and data extraction with AH and AD. All authors participated in the full-text review process, manuscript drafting, the critical revision of the content, and approved the final manuscript.

## CRediT authorship contribution statement

**Binyam Minuye Birhane:** Writing – original draft, Data curation, Writing – review & editing, Methodology, Conceptualization. **Angela Dawson:** Visualization, Methodology, Conceptualization, Writing – review & editing, Supervision, Data curation. **Andrew Hayen:** Writing – review & editing, Validation, Methodology, Conceptualization, Visualization, Supervision, Data curation.

## Declaration of competing interest

The authors declare that they have no known competing financial interests or personal relationships that could have appeared to influence the work reported in this paper.

## Appendix Supplementary materials

Image, application 1

Image, application 2

Image, application 3
