# Supplementary material for: Health of refugee children upon arrival in high-income countries: A scoping review
Source: J Migr Health. 2025 Oct 29;12:100373. doi: 10.1016/j.jmh.2025.100373 (PMC12664805; doi:10.1016/j.jmh.2025.100373)
Supplement: Supplementary file 2 [file mmc2.docx]

Appendix 2: Search history from databases

1. EMBASE

| # | Query | Results |
| --- | --- | --- |
| 1 | child/ or child health/ or child growth/ | 2,110,941 |
| 2 | adolescent/ | 1,754,479 |
| 3 | pediatrics/ | 93,000 |
| 4 | infant/ | 692,840 |
| 5 | newborn/ or neonat*.mp. | 791,336 |
| 6 | 1 or 2 or 3 or 4 or 5 | 3,944,924 |
| 7 | cancer mortality/ or childhood mortality/ or infant mortality/ or newborn mortality/ or mortality/ or perinatal mortality/ | 1,047,588 |
| 8 | morbidity.mp. or morbidity/ or newborn morbidity/ | 812,658 |
| 9 | child health/ | 35,640 |
| 10 | disability-adjusted life year/ | 4,304 |
| 11 | disability/cn, di, ep [Congenital Disorder, Diagnosis, Epidemiology] | 1,926 |
| 12 | anthropometry/ | 66,844 |
| 13 | protein calorie malnutrition/ or Malnutrition Universal Screening Tool/ or malnutrition/ or malnutrition assessment/ | 79,959 |
| 14 | stunting/ep [Epidemiology] | 351 |
| 15 | obesity/ or childhood obesity/ | 544,844 |
| 16 | diseases/cn, di, ep [Congenital Disorder, Diagnosis, Epidemiology] | 252 |
| 17 | "anemia of chronic disease"/ or anemia/ or iron deficiency anemia/ | 251,270 |
| 18 | asthma/ep [Epidemiology] | 12,557 |
| 19 | injury/ep [Epidemiology] | 8,487 |
| 20 | accident/pc [Prevention] | 2 |
| 21 | pneumonia/ep [Epidemiology] | 5,045 |
| 22 | respiratory tract infection/ep [Epidemiology] | 4,535 |
| 23 | diarrhea/ or acute diarrhea/ or bloody diarrhea/ or chronic diarrhea/ | 293,537 |
| 24 | malaria/ or insecticide treated net/ or bed net/ | 83,965 |
| 25 | vaccination/ or measles vaccination/ or influenza vaccination/ or vaccination coverage/ | 230,116 |
| 26 | diphtheria pertussis tetanus vaccine/ct [Clinical Trial] | 470 |
| 27 | Quality of life.mp. or "quality of life"/ | 784,887 |
| 28 | Acute condition.mp. or acute disease/ | 98,661 |
| 29 | communicable disease/ or obesity/ or non-communicable disease/ or cardiovascular disease/ or chronic disease/ or diabetes mellitus/ or hypertension/ | 2,035,991 |
| 30 | childhood cancer/ep [Epidemiology] | 2,359 |
| 31 | Immunisation.mp. or immunization/ | 120,294 |
| 32 | child death/ or newborn death/ or "cause of death"/ or "time of death"/ or death/ | 439,159 |
| 33 | mental health/ or wellbeing/ | 276,490 |
| 34 | health status indicator/ | 3,446 |
| 35 | 7 or 8 or 9 or 10 or 11 or 12 or 13 or 14 or 15 or 16 or 17 or 18 or 19 or 20 or 21 or 22 or 23 or 24 or 25 or 26 or 27 or 28 or 29 or 30 or 31 or 32 or 33 or 34 | 5,435,861 |
| 36 | refugee camp/ or refugee/ | 16,951 |
| 37 | asylum seeker/ or "asylum (legal)"/ or asylum seeker center/ | 1,639 |
| 38 | migration/ | 40,879 |
| 39 | 36 or 37 or 38 | 56,624 |
| 40 | Scandinavia/ep [Epidemiology] | 187 |
| 41 | high income country/ | 8,580 |
| 42 | (((Andorra or (Antigua and Barbuda) or Aruba or Australia or Austria or Bahamas or Bahrain or Barbados or Belgium or Bermuda or British Virgin Islands or Brunei Darussalam or Canada or Cayman Islands or Channel Islands or Chile or Croatia or Curacao or Cyprus or Czech Republic or Denmark or Estonia or Equatorial Guinea or Faroe Islands or Finland or France or French Polynesia or Germany or Gibraltar or Greece or Greenland or Guam or Hong Kong SAR or Hungary or Iceland or Ireland or Isle Man or Israel or Italy or Japan or Korea or Kuwait or Latvia or Liechtenstein or Lithuania or Luxembourg or Macao SAR or Malta or Mauritius or Monaco or Montenegro or Netherlands or New Caledonia or New Zealand or Northern Mariana Islands or Norway or Oman or Palau or Panama or Poland or Portugal or Puerto Rico or Qatar or Romania or Russian Federation or San Marino or Saudi Arabia or Seychelles or Singapore or Sint Maarten or Slovak Republic or Slovenia or Spain or Saint Kitts) and Nevis) or Saint Martin or Sweden or Switzerland or Taiwan or (Trinidad and Tobago) or (Turks and Caicos Islands) or United Arab Emirates or United Kingdom or United States or Uruguay or Virgin Islands).mp. [mp=title, abstract, heading word, drug trade name, original title, device manufacturer, drug manufacturer, device trade name, keyword heading word, floating subheading word, candidate term word] | 3,048,562 |
| 43 | 40 or 41 or 42 | 3,056,104 |
| 44 | 6 and 35 and 39 and 43 | 757 |

1. MEDLINE

| # | Query | Results |
| --- | --- | --- |
| 1 | child/ or child health/ or child growth/ | 1,902,817 |
| 2 | adolescent/ | 2,209,313 |
| 3 | pediatrics/ | 57,813 |
| 4 | infant/ | 862,952 |
| 5 | newborn/ or neonat*.mp. | 815,669 |
| 6 | 1 or 2 or 3 or 4 or 5 | 3,980,627 |
| 7 | cancer mortality/ or childhood mortality/ or infant mortality/ or newborn mortality/ or mortality/ or perinatal mortality/ | 78,314 |
| 8 | morbidity.mp. or morbidity/ or newborn morbidity/ | 461,658 |
| 9 | child health/ | 4,928 |
| 10 | disability-adjusted life year/ | 176 |
| 11 | disability/cn, di, ep [Congenital Disorder, Diagnosis, Epidemiology] | 0 |
| 12 | anthropometry/ | 41,237 |
| 13 | protein calorie malnutrition/ or Malnutrition Universal Screening Tool/ or malnutrition/ or malnutrition assessment/ | 26,190 |
| 14 | stunting/ep [Epidemiology] | 2,683 |
| 15 | obesity/ or childhood obesity/ | 226,786 |
| 16 | diseases/cn, di, ep [Congenital Disorder, Diagnosis, Epidemiology] | 11 |
| 17 | "anemia of chronic disease"/ or anemia/ or iron deficiency anemia/ | 64,873 |
| 18 | asthma/ep [Epidemiology] | 19,748 |
| 19 | injury/ep [Epidemiology] | 14,411 |
| 20 | accident/pc [Prevention] | 166 |
| 21 | pneumonia/ep [Epidemiology] | 5,914 |
| 22 | respiratory tract infection/ep [Epidemiology] | 9,231 |
| 23 | diarrhea/ or acute diarrhea/ or bloody diarrhea/ or chronic diarrhea/ | 52,272 |
| 24 | malaria/ or insecticide treated net/ or bed net/ | 50,729 |
| 25 | vaccination/ or measles vaccination/ or influenza vaccination/ or vaccination coverage/ | 105,037 |
| 26 | [diphtheria pertussis tetanus vaccine/ct [Clinical Trial]] | 0 |
| 27 | Quality of life.mp. or "quality of life"/ | 443,207 |
| 28 | Acute condition.mp. or acute disease/ | 223,121 |
| 29 | communicable disease/ or obesity/ or non-communicable disease/ or cardiovascular disease/ or chronic disease/ or diabetes mellitus/ or hypertension/ | 1,017,358 |
| 30 | childhood cancer/ep [Epidemiology] | 0 |
| 31 | Immunisation.mp. or immunization/ | 63,251 |
| 32 | child death/ or newborn death/ or "cause of death"/ or "time of death"/ or death/ | 72,869 |
| 33 | mental health/ or wellbeing/ | 60,023 |
| 34 | health status indicator/ | 24,085 |
| 35 | 7 or 8 or 9 or 10 or 11 or 12 or 13 or 14 or 15 or 16 or 17 or 18 or 19 or 20 or 21 or 22 or 23 or 24 or 25 or 26 or 27 or 28 or 29 or 30 or 31 or 32 or 33 or 34 | 2,606,563 |
| 36 | refugee camp/ or refugee/ | 13,153 |
| 37 | asylum seeker/ or "asylum (legal)"/ or asylum seeker center/ | 13,046 |
| 38 | migration/ | 0 |
| 39 | 36 or 37 or 38 | 13,153 |
| 40 | Scandinavia/ep [Epidemiology] | 1,109 |
| 41 | High-income country/ | 0 |
| 42 | (((Andorra or (Antigua and Barbuda) or Aruba or Australia or Austria or Bahamas or Bahrain or Barbados or Belgium or Bermuda or British Virgin Islands or Brunei Darussalam or Canada or Cayman Islands or Channel Islands or Chile or Croatia or Curacao or Cyprus or Czech Republic or Denmark or Estonia or Equatorial Guinea or Faroe Islands or Finland or France or French Polynesia or Germany or Gibraltar or Greece or Greenland or Guam or Hong Kong SAR or Hungary or Iceland or Ireland or Isle Man or Israel or Italy or Japan or Korea or Kuwait or Latvia or Liechtenstein or Lithuania or Luxembourg or Macao SAR or Malta or Mauritius or Monaco or Montenegro or Netherlands or New Caledonia or New Zealand or Northern Mariana Islands or Norway or Oman or Palau or Panama or Poland or Portugal or Puerto Rico or Qatar or Romania or Russian Federation or San Marino or Saudi Arabia or Seychelles or Singapore or Sint Maarten or Slovak Republic or Slovenia or Spain or Saint Kitts) and Nevis) or Saint Martin or Sweden or Switzerland or Taiwan or (Trinidad and Tobago) or (Turks and Caicos Islands) or United Arab Emirates or United Kingdom or United States or Uruguay or Virgin Islands).mp. [mp=title, book title, abstract, original title, name of substance word, subject heading word, floating sub-heading word, keyword heading word, organism supplementary concept word, protocol supplementary concept word, rare disease supplementary concept word, unique identifier, synonyms, population supplementary concept word, anatomy supplementary concept word] | 1,621,650 |
| 43 | 40 or 41 or 42 | 1,622,280 |
| 44 | 6 and 35 and 39 and 43 | 243 |

1. Emcare

| # | Query | Results |
| --- | --- | --- |
| 1 | child/ or child health/ or child growth/ | 475,869 |
| 2 | adolescent/ | 306,496 |
| 3 | pediatrics/ | 23,148 |
| 4 | infant/ | 122,766 |
| 5 | newborn/ or neonat*.mp. | 138,059 |
| 6 | 1 or 2 or 3 or 4 or 5 | 802,635 |
| 7 | cancer mortality/ or childhood mortality/ or infant mortality/ or newborn mortality/ or mortality/ or perinatal mortality/ | 189,931 |
| 8 | morbidity.mp. or morbidity/ or newborn morbidity/ | 181,452 |
| 9 | child health/ | 16,218 |
| 10 | disability-adjusted life year/ | 763 |
| 11 | [disability/cn, di, ep [Congenital Disorder, Diagnosis, Epidemiology]] | 0 |
| 12 | anthropometry/ | 15,047 |
| 13 | protein calorie malnutrition/ or Malnutrition Universal Screening Tool/ or malnutrition/ or malnutrition assessment/ | 19,328 |
| 14 | [stunting/ep [Epidemiology]] | 0 |
| 15 | obesity/ or childhood obesity/ | 113,105 |
| 16 | [diseases/cn, di, ep [Congenital Disorder, Diagnosis, Epidemiology]] | 0 |
| 17 | "anemia of chronic disease"/ or anemia/ or iron deficiency anemia/ | 34,575 |
| 18 | [asthma/ep [Epidemiology]] | 0 |
| 19 | [injury/ep [Epidemiology]] | 0 |
| 20 | [accident/pc [Prevention]] | 0 |
| 21 | [pneumonia/ep [Epidemiology]] | 0 |
| 22 | [respiratory tract infection/ep [Epidemiology]] | 0 |
| 23 | diarrhea/ or acute diarrhea/ or bloody diarrhea/ or chronic diarrhea/ | 43,225 |
| 24 | malaria/ or insecticide treated net/ or bed net/ | 12,051 |
| 25 | vaccination/ or measles vaccination/ or influenza vaccination/ or vaccination coverage/ | 32,862 |
| 26 | [diphtheria pertussis tetanus vaccine/ct [Clinical Trial]] | 0 |
| 27 | Quality of life.mp. or "quality of life"/ | 224,787 |
| 28 | Acute condition.mp. or acute disease/ | 12,301 |
| 29 | communicable disease/ or obesity/ or non communicable disease/ or cardiovascular disease/ or chronic disease/ or diabetes mellitus/ or hypertension/ | 361,462 |
| 30 | [childhood cancer/ep [Epidemiology]] | 0 |
| 31 | Immunisation.mp. or immunization/ | 17,644 |
| 32 | child death/ or newborn death/ or "cause of death"/ or "time of death"/ or death/ | 102,165 |
| 33 | mental health/ or wellbeing/ | 148,172 |
| 34 | health status indicator/ | 423 |
| 35 | 7 or 8 or 9 or 10 or 11 or 12 or 13 or 14 or 15 or 16 or 17 or 18 or 19 or 20 or 21 or 22 or 23 or 24 or 25 or 26 or 27 or 28 or 29 or 30 or 31 or 32 or 33 or 34 | 1,144,487 |
| 36 | refugee camp/ or refugee/ | 7,056 |
| 37 | asylum seeker/ or "asylum (legal)"/ or asylum seeker center/ | 1,002 |
| 38 | migration/ | 3,129 |
| 39 | 36 or 37 or 38 | 10,749 |
| 40 | [Scandinavia/ep [Epidemiology]] | 0 |
| 41 | high income country/ | 1,767 |
| 42 | (((Andorra or (Antigua and Barbuda) or Aruba or Australia or Austria or Bahamas or Bahrain or Barbados or Belgium or Bermuda or British Virgin Islands or Brunei Darussalam or Canada or Cayman Islands or Channel Islands or Chile or Croatia or Curacao or Cyprus or Czech Republic or Denmark or Estonia or Equatorial Guinea or Faroe Islands or Finland or France or French Polynesia or Germany or Gibraltar or Greece or Greenland or Guam or Hong Kong SAR or Hungary or Iceland or Ireland or Isle Man or Israel or Italy or Japan or Korea or Kuwait or Latvia or Liechtenstein or Lithuania or Luxembourg or Macao SAR or Malta or Mauritius or Monaco or Montenegro or Netherlands or New Caledonia or New Zealand or Northern Mariana Islands or Norway or Oman or Palau or Panama or Poland or Portugal or Puerto Rico or Qatar or Romania or Russian Federation or San Marino or Saudi Arabia or Seychelles or Singapore or Sint Maarten or Slovak Republic or Slovenia or Spain or Saint Kitts) and Nevis) or Saint Martin or Sweden or Switzerland or Taiwan or (Trinidad and Tobago) or (Turks and Caicos Islands) or United Arab Emirates or United Kingdom or United States or Uruguay or Virgin Islands).mp. [mp=title, abstract, heading word, drug trade name, original title, device manufacturer, drug manufacturer, device trade name, keyword heading word] | 763,625 |
| 43 | 40 or 41 or 42 | 765,157 |
| 44 | 6 and 35 and 39 and 43 | 201 |

1. CINHAL

| # | Query | Results | |
| --- | --- | --- | --- |
| S53 | S7 AND S36 AND S39 AND S52 | 223 |  |
| S52 | S40 OR S41 OR S42 OR S43 OR S44 OR S45 OR S46 OR S47 OR S48 OR S49 OR S50 OR S51 | 403,907 | |
| S51 | (MH "United Arab Emirates") OR (MH "United States Agency for Healthcare Research and Quality") OR (MH "United States Department of Health and Human Services") OR (MH "United States Public Health Service") OR (MH "United States by Individual State") OR "United Arab Emirates OR United Kingdom OR United States OR Uruguay OR Virgin Islands" OR (MH "Virgin Islands of the United States") | 10,541 | |
| S50 | (MH "Switzerland") OR (MH "Taiwan") OR (MH "Sweden") OR (MH "Trinidad and Tobago") OR "Nevis OR Saint Martin OR Sweden OR Switzerland OR Taiwan OR (Trinidad and Tobago) OR (Turks and Caicos Islands)" | 63,749 | |
| S49 | (MH "Saudi Arabia") OR (MH "San Marino") OR (MH "Singapore") OR (MH "Spain") OR (MH "Slovakia") OR (MH "Slovenia") | 42,953 | |
| S48 | (MH "Oman") OR (MH "Poland") OR (MH "Panama") OR (MH "Portugal") OR (MH "Qatar") OR (MH "Romania") OR "Oman OR Palau OR Panama OR Poland OR Portugal OR Puerto Rico Qatar OR Romania OR Russian Federation" | 18,284 | |
| S47 | (MH "New Zealand") OR "Malta OR Mauritius OR Monaco OR Montenegro OR Netherlands OR New Caledonia OR New Zealand OR Northern Mariana Islands OR Norway" | 31,413 | |
| S46 | (MH "South Korea") OR (MH "North Korea") OR (MH "Kuwait") OR (MH "Japan") OR (MH "Korea") OR (MH "Liechtenstein") OR (MH "Latvia") OR (MH "Lithuania") OR (MH "Luxembourg") OR (MH "Macao") | 64,607 | |
| S45 | (MH "Hong Kong") OR (MH "Germany, West") OR (MH "Germany, East") OR (MH "Ireland") OR (MH "Greece") OR "Germany OR Gibraltar OR Greece OR Greenland OR Guam OR Hong Kong SAR OR Hungary OR Iceland OR Ireland OR Isle Man OR Israel OR Italy" | 36,284 | |
| S44 | (MH "Denmark") OR (MH "Finland") OR (MH "Equatorial Guinea") OR (MH "Polynesia") | 32,727 | |
| S43 | (MH "Czech Republic") OR "Canada OR Cayman Islands OR Channel Islands OR Chile OR Croatia OR Curacao OR Cyprus OR Czech Republic" "Bahamas OR Bahrain OR Barbados OR Belgium OR Bermuda OR British Virgin Islands OR Brunei Darussalam" OR (MH "Virgin Islands of the United States" | 2,206 | |
| S42 | "Andorra OR Antigua and Barbuda OR Aruba OR Australia OR Austria OR Bahamas OR Bahrain OR Barbados OR Belgium OR Bermuda OR British Virgin Islands OR Brunei Darussalam" OR "Andorra OR Antigua and Barbuda OR Aruba OR Australia OR Austria OR Bahamas OR Bahrain OR Barbados OR Belgium OR Bermuda OR British Virgin Islands OR Brunei Darussalam OR Canada OR Cayman Islands OR Channel Islands OR Chile OR Croatia OR Curacao OR Cyprus OR Czech Republic" OR (MH "Austria") OR (MH "Andorra") OR (MH "Australia") OR (MH "Antigua") OR "Andorra OR Antigua and Barbuda OR Aruba OR Australia OR Austria" | 90,908 | |
| S41 | (MH "Sweden") OR (MH "Norway") OR (MH "Finland") OR (MH "Denmark") OR (MH "Iceland") OR (MH "Greenland") | 81,160 | |
| S40 | "high income countries" OR (MH "Developed Countries") | 8,212 | |
| S39 | S37 OR S38 | 24,131 | |
| S38 | (MH "Camps") | 1,711 | |
| S37 | (MH "Refugees") OR (MH "Refugee Camps") OR (MH "Transients and Migrants") OR (MH "Emigration and Immigration") OR (MH "Orphans and Orphanages") OR (MH "Undocumented Immigrants") | 22,508 | |
| S36 | S8 OR S9 OR S10 OR S11 OR S12 OR S13 OR S14 OR S15 OR S16 OR S17 OR S18 OR S19 OR S20 OR S21 OR S22 OR S23 OR S24 OR S25 OR S26 OR S27 OR S28 OR S29 OR S30 OR S31 OR S32 OR S33 OR S34 OR S35 | 1,019,279 | |
| S35 | (MH "Life Course Perspective") OR (MH "Healthy Life Expectancy") OR (MH "Life Expectancy") | 9,241 | |
| S34 | (MH "Health Status") OR (MH "Health Status Indicators") OR (MH "Well-Being (Iowa NOC)") OR (MH "Health Status Disparities") | 82,047 | |
| S33 | (MH "Life Experiences") OR (MH "Adverse Childhood Experiences") | 37,274 | |
| S32 | (MH "Hospitalization") OR (MH "Hospitalization of Older Persons") OR (MH "Hospitals, Pediatric") | 60,225 | |
| S31 | (MH "Childhood Neoplasms") OR (MH "Hematologic Neoplasms") | 9,611 | |
| S30 | (MH "Quality of Life") OR (MH "Well-Being (Iowa NOC)") | 138,356 | |
| S29 | (MH "Vaccination Status") OR (MH "Immunization") OR (MH "Diphtheria-Tetanus-Pertussis Vaccine") OR (MH "Measles-Mumps-Rubella-Varicella Vaccine") | 29,596 | |
| S28 | (MH "Mosquito Nets") | 69 | |
| S27 | (MH "Accidents") | 3,368 | |
| S26 | (MH "Death") OR (MH "Perinatal Death") OR (MH "Cause of Death") | 44,170 | |
| S25 | (MH "Diarrhea") OR (MH "Diarrhea (Saba CCC)") OR (MH "Diarrhea Management (Iowa NIC)") OR (MH "Rehydration Solutions") OR (MH "Diarrhea Care (Saba CCC)") | 12,169 | |
| S24 | (MH "Pneumonia") | 12,526 | |
| S23 | (MH "Middle East Respiratory Syndrome") OR (MH "Respiratory Tract Infections+") | 91,338 | |
| S22 | (MH "Asthma") | 36,462 | |
| S21 | (MH "Anemia") | 11,423 | |
| S20 | (MH "Trauma") | 18,432 | |
| S19 | (MH "National Alliance on Mental Illness") OR (MH "Treatment Behavior: Illness or Injury (Iowa NOC)") OR (MH "Psychosocial Aspects of Illness") | 4,179 | |
| S18 | (MH "Child Development Disorders+") OR (MH "Mental Disorders Diagnosed in Childhood+") OR (MH "Child Development Disorders, Pervasive+") OR (MH "Child Nutrition Disorders+") OR (MH "Child Behavior Disorders+") | 107,346 | |
| S17 | (MH "Disease") | 8,385 | |
| S16 | (MH "Overnutrition") | 48 | |
| S15 | (MH "Pediatric Obesity") OR (MH "Pickwickian Syndrome") OR (MH "Obesity+") | 112,653 | |
| S14 | (MH "Undernutrition") | 130 | |
| S13 | (MH "Malnutrition+") OR (MH "Protein-Energy Malnutrition") | 12,648 | |
| S12 | (MH "Anthropometry") | 13,618 | |
| S11 | (MH "Quality-Adjusted Life Years") OR (MH "Healthy Life Expectancy") OR (MH "Disability-Adjusted Life Years") | 6,377 | |
| S10 | (MH "Child Health") OR (MH "Child Abuse, Sexual") OR (MH "Child Behavior Disorders+") OR (MH "Child Abuse Survivors") | 35,775 | |
| S9 | (MH "Morbidity+") OR (MH "Comorbidity") OR (MH "Healthy Life Expectancy") OR (MH "Health Status Indicators+") | 261,834 | |
| S8 | (MH "Hospital Mortality") OR (MH "Child Mortality") OR (MH "Infant Mortality") OR (MH "Mortality+") | 82,330 | |
| S7 | S1 OR S2 OR S3 OR S4 OR S5 OR S6 | 735,541 | |
| S6 | (MH "Infant, Newborn+") OR (MH "Infant, Newborn, Diseases+") | 172,830 | |
| S5 | (MH "Infant+") OR (MH "Infant, Premature") OR (MH "Infant, High Risk") OR (MH "Infant Death+") OR (MH "Infant, Very Low Birth Weight") OR (MH "Infant, Small for Gestational Age") OR (MH "Infant, Large for Gestational Age") OR (MH "Infant, Newborn, Diseases+") OR (MH "Infant Behavior") | 300,464 | |
| S4 | (MH "Pediatric Obesity") | 16,767 | |
| S3 | (MM "Adolescence") OR (MM "Adolescent Health") | 4,711 | |
| S2 | (MH "Child Mortality") | 2,915 | |
| S1 | (MH "Child") OR (MH "Children with Disabilities") OR (MH "Child Abuse") | 530,929 | |

1. SCOPUS

((TITLE-ABS-KEY(child*)) OR (TITLE-ABS-KEY(adolescen*)) OR (TITLE-ABS-KEY(Paediatric*)) OR (TITLE-ABS-KEY(pediatric*)) OR (TITLE-ABS-KEY(infant*)) OR (TITLE-ABS-KEY(neonat*))) AND (TITLE-ABS-KEY(mortality OR morbidity OR "Child health" OR "Disability- adjusted life years" OR disability OR anthropometry OR malnutrition OR stunting OR obesity OR disease OR illness OR disorder OR anaemia OR anemia OR asthma OR infection OR pneumonia OR diarrhea OR trauma OR injury OR death OR accident OR "Insecticide-treated net" OR immunization OR vaccin* OR "diphtheria pertussis tetanus" OR " health condition" OR " quality of life " OR "Childhood cancer" OR hospitalization OR wellbeing OR "mental health" OR "physical health" OR "Health status indicator" OR "Life experience")) AND (TITLE-ABS-KEY(refugee OR "refugee camp" OR camp OR immigration OR asylum OR migrant* OR "displaced people camp")) AND ((TITLE-ABS-KEY("Scandinavian countr*")) OR (TITLE-ABS-KEY("high income countr*")) OR (TITLE-ABS-KEY(Sweden OR Norway OR Finland OR Denmark OR Iceland OR Greenland)) OR (TITLE-ABS-KEY(Denmark OR Estonia OR "Equatorial Guinea" OR "Faroe Islands" OR Finland OR France OR "French Polynesia" OR Germany OR Gibraltar OR Greece OR Greenland OR Guam OR "Hong Kong SAR" OR Hungary)) OR (TITLE-ABS-KEY(Iceland OR Ireland OR "Isle Man" OR Israel OR Italy OR Japan OR Korea OR Kuwait OR Latvia OR Liechtenstein OR Lithuania OR Luxembourg OR "Macao SAR" OR Malta OR Mauritius OR Monaco OR Montenegro OR Netherlands OR "New Caledonia" OR "New Zealand" OR "Northern Mariana Islands" OR Norway)) OR (TITLE-ABS-KEY(oman OR palau OR panama OR poland OR portugal OR "Puerto Rico" OR qatar OR romania OR "Russian Federation" OR "San Marino" OR "Saudi Arabia" OR seychelles OR singapore OR "Sint Maarten" OR "Slovak Republic" OR slovenia OR spain OR "saint kitts and nevis" OR "saint and martin" OR sweden OR switzerland OR taiwan OR "trinidad and tobago" OR "turks caicos and islands" OR "United Arab Emirates" OR "United Kingdom" OR "United States" OR uruguay OR "Virgin Islands ")) OR (TITLE-ABS-KEY(andorra OR "antigua and barbuda" OR aruba OR australia OR austria OR bahamas OR bahrain OR barbados OR belgium OR berandorra OR "British Virgin Islands" OR "Brunei Darussalam" OR canada OR "Cayman Islands" OR "Channel Islands" OR chile OR croatia OR curacao OR cyprus OR "Czech Republic"))) AND ( EXCLUDE ( SRCTYPE,"d" ) OR EXCLUDE ( SRCTYPE,"k" ) OR EXCLUDE ( SRCTYPE,"b" ) OR EXCLUDE ( SRCTYPE,"Undefined" ) ) AND ( LIMIT-TO ( AFFILCOUNTRY,"United States" ) OR LIMIT-TO ( AFFILCOUNTRY,"United Kingdom" ) OR LIMIT-TO ( AFFILCOUNTRY,"Australia" ) OR LIMIT-TO ( AFFILCOUNTRY,"Canada" ) OR LIMIT-TO ( AFFILCOUNTRY,"Germany" ) OR LIMIT-TO ( AFFILCOUNTRY,"Sweden" ) OR LIMIT-TO ( AFFILCOUNTRY,"Spain" ) OR LIMIT-TO ( AFFILCOUNTRY,"Italy" ) OR LIMIT-TO ( AFFILCOUNTRY,"Netherlands" ) OR LIMIT-TO ( AFFILCOUNTRY,"France" ) OR LIMIT-TO ( AFFILCOUNTRY,"Norway" ) OR LIMIT-TO ( AFFILCOUNTRY,"Israel" ) OR LIMIT-TO ( AFFILCOUNTRY,"Denmark" ) OR LIMIT-TO ( AFFILCOUNTRY,"Switzerland" ) OR LIMIT-TO ( AFFILCOUNTRY,"Belgium" ) OR LIMIT-TO ( AFFILCOUNTRY,"Greece" ) OR LIMIT-TO ( AFFILCOUNTRY,"New Zealand" ) OR LIMIT-TO ( AFFILCOUNTRY,"Finland" ) OR LIMIT-TO ( AFFILCOUNTRY,"Austria" ) OR LIMIT-TO ( AFFILCOUNTRY,"Ireland" ) OR LIMIT-TO ( AFFILCOUNTRY,"Japan" ) OR LIMIT-TO ( AFFILCOUNTRY,"South Korea" ) OR LIMIT-TO ( AFFILCOUNTRY,"Mexico" ) OR LIMIT-TO ( AFFILCOUNTRY,"Chile" ) OR LIMIT-TO ( AFFILCOUNTRY,"Poland" ) OR LIMIT-TO ( AFFILCOUNTRY,"China" ) OR LIMIT-TO ( AFFILCOUNTRY,"Croatia" ) OR LIMIT-TO ( AFFILCOUNTRY,"Portugal" ) OR LIMIT-TO ( AFFILCOUNTRY,"Taiwan" ) OR LIMIT-TO ( AFFILCOUNTRY,"Turkey" ) OR LIMIT-TO ( AFFILCOUNTRY,"Russian Federation" ) OR LIMIT-TO ( AFFILCOUNTRY,"Singapore" ) OR LIMIT-TO ( AFFILCOUNTRY,"Saudi Arabia" ) OR LIMIT-TO ( AFFILCOUNTRY,"Qatar" ) OR LIMIT-TO ( AFFILCOUNTRY,"Hong Kong" ) OR LIMIT-TO ( AFFILCOUNTRY,"Hungary" ) OR LIMIT-TO ( AFFILCOUNTRY,"Czech Republic" ) OR LIMIT-TO ( AFFILCOUNTRY,"United Arab Emirates" ) OR LIMIT-TO ( AFFILCOUNTRY,"Viet Nam" ) OR LIMIT-TO ( AFFILCOUNTRY,"Iceland" ) OR LIMIT-TO ( AFFILCOUNTRY,"Pakistan" ) OR LIMIT-TO ( AFFILCOUNTRY,"Kuwait" ) OR LIMIT-TO ( AFFILCOUNTRY,"Malta" ) OR LIMIT-TO ( AFFILCOUNTRY,"Romania" ) OR LIMIT-TO ( AFFILCOUNTRY,"Iraq" ) OR LIMIT-TO ( AFFILCOUNTRY,"Ukraine" ) OR LIMIT-TO ( AFFILCOUNTRY,"Cote d'Ivoire" ) OR LIMIT-TO ( AFFILCOUNTRY,"Brunei Darussalam" ) OR LIMIT-TO ( AFFILCOUNTRY,"Germany (Democratic Republic, DDR)" ) OR LIMIT-TO ( AFFILCOUNTRY,"Greenland" ) OR LIMIT-TO ( AFFILCOUNTRY,"Netherlands Antilles" ) OR LIMIT-TO ( AFFILCOUNTRY,"New Caledonia" ) OR LIMIT-TO ( AFFILCOUNTRY,"Panama" ) OR LIMIT-TO ( AFFILCOUNTRY,"Trinidad and Tobago" ) OR LIMIT-TO ( AFFILCOUNTRY,"Uruguay" ) OR LIMIT-TO ( AFFILCOUNTRY,"Virgin Islands (British)" ) OR EXCLUDE ( AFFILCOUNTRY,"South Africa" ) OR EXCLUDE ( AFFILCOUNTRY,"Thailand" ) OR EXCLUDE ( AFFILCOUNTRY,"Brazil" ) OR EXCLUDE ( AFFILCOUNTRY,"Kenya" ) OR EXCLUDE ( AFFILCOUNTRY,"Colombia" ) OR EXCLUDE ( AFFILCOUNTRY,"India" ) OR EXCLUDE ( AFFILCOUNTRY,"Pakistan" ) OR EXCLUDE ( AFFILCOUNTRY,"Cameroon" ) OR EXCLUDE ( AFFILCOUNTRY,"Egypt" ) OR EXCLUDE ( AFFILCOUNTRY,"Indonesia" ) OR EXCLUDE ( AFFILCOUNTRY,"Bangladesh" ) OR EXCLUDE ( AFFILCOUNTRY,"Benin" ) OR EXCLUDE ( AFFILCOUNTRY,"Bolivia" ) OR EXCLUDE ( AFFILCOUNTRY,"Bosnia and Herzegovina" ) OR EXCLUDE ( AFFILCOUNTRY,"Botswana" ) OR EXCLUDE ( AFFILCOUNTRY,"Sri Lanka" ) OR EXCLUDE ( AFFILCOUNTRY,"Venezuela" ) OR EXCLUDE ( AFFILCOUNTRY,"Algeria" ) OR EXCLUDE ( AFFILCOUNTRY,"Bahrain" ) OR EXCLUDE ( AFFILCOUNTRY,"Bhutan" ) OR EXCLUDE ( AFFILCOUNTRY,"Burkina Faso" ) OR EXCLUDE ( AFFILCOUNTRY,"Cambodia" ) OR EXCLUDE ( AFFILCOUNTRY,"Chad" ) OR EXCLUDE ( AFFILCOUNTRY,"Congo" ) OR EXCLUDE ( AFFILCOUNTRY,"Faroe Islands" ) OR EXCLUDE ( AFFILCOUNTRY,"Gabon" ) OR EXCLUDE ( AFFILCOUNTRY,"Gambia" ) OR EXCLUDE ( AFFILCOUNTRY,"Sierra Leone" ) OR EXCLUDE ( AFFILCOUNTRY,"Somalia" ) OR EXCLUDE ( AFFILCOUNTRY,"Tanzania" ) ) AND ( LIMIT-TO ( DOCTYPE,"ar" ) OR LIMIT-TO ( DOCTYPE,"re" ) ) AND ( LIMIT-TO ( SUBJAREA,"MEDI" ) OR LIMIT-TO ( SUBJAREA,"PSYC" ) OR LIMIT-TO ( SUBJAREA,"NURS" ) OR EXCLUDE ( SUBJAREA,"AGRI" ) OR EXCLUDE ( SUBJAREA,"ARTS" ) OR EXCLUDE ( SUBJAREA,"BIOC" ) OR EXCLUDE ( SUBJAREA,"BUSI" ) OR EXCLUDE ( SUBJAREA,"CHEM" ) OR EXCLUDE ( SUBJAREA,"COMP" ) OR EXCLUDE ( SUBJAREA,"DECI" ) OR EXCLUDE ( SUBJAREA,"DENT" ) OR EXCLUDE ( SUBJAREA,"EART" ) OR EXCLUDE ( SUBJAREA,"ECON" ) OR EXCLUDE ( SUBJAREA,"IMMU" ) OR EXCLUDE ( SUBJAREA,"NEUR" ) OR EXCLUDE ( SUBJAREA,"PHAR" ) OR EXCLUDE ( SUBJAREA,"ENGI" ) OR EXCLUDE ( SUBJAREA,"ENVI" ) OR EXCLUDE ( SUBJAREA,"PHYS" ) OR EXCLUDE ( SUBJAREA,"VETE" ) OR EXCLUDE ( SUBJAREA,"SOCI" ) ) AND ( EXCLUDE ( DOCTYPE,"re" ) ) AND ( EXCLUDE ( EXACTKEYWORD,"Questionnaires" ) ) AND ( EXCLUDE ( EXACTKEYWORD,"Acculturation" ) ) AND ( EXCLUDE ( EXACTKEYWORD,"Geographic Distribution" ) OR EXCLUDE ( EXACTKEYWORD,"Procedures" ) ) AND ( EXCLUDE ( AFFILCOUNTRY,"Ethiopia" ) OR EXCLUDE ( AFFILCOUNTRY,"Palestine" ) OR EXCLUDE ( AFFILCOUNTRY,"Sudan" ) ) AND ( EXCLUDE ( AFFILCOUNTRY,"Malaysia" ) ) AND ( EXCLUDE ( EXACTSRCTITLE,"Swiss Medical Weekly" ) OR EXCLUDE ( EXACTSRCTITLE,"Australian Family Physician" ) ) AND ( EXCLUDE ( SUBJAREA,"HEAL" ) ) AND ( EXCLUDE ( EXACTSRCTITLE,"Enfermedades Infecciosas Y Microbiologia Clinica" ) ) AND ( EXCLUDE ( EXACTKEYWORD,"Middle Aged" ) ) AND ( EXCLUDE ( EXACTKEYWORD,"Surveys And Questionnaires" ) ) AND ( EXCLUDE ( EXACTKEYWORD,"Case Report" ) ) AND ( EXCLUDE ( EXACTKEYWORD,"Aged" ) ) AND ( EXCLUDE ( EXACTKEYWORD,"Health Care Policy" ) ) AND ( EXCLUDE ( EXACTKEYWORD,"Cultural Anthropology" ) ) AND ( EXCLUDE ( EXACTKEYWORD,"Africa" ) ) AND ( EXCLUDE ( EXACTKEYWORD,"Attitude To Health" ) ) AND ( EXCLUDE ( EXACTKEYWORD,"Education" ) ) AND ( EXCLUDE ( EXACTKEYWORD,"Educational Status" ) ) AND ( EXCLUDE ( EXACTKEYWORD,"Socioeconomics" ) ) AND ( EXCLUDE ( EXACTKEYWORD,"Demography" ) ) AND ( EXCLUDE ( EXACTKEYWORD,"Cultural Factor" ) ) AND ( EXCLUDE ( EXACTKEYWORD,"Practice Guideline" ) ) **Result: 962**

1. WEB OF SCIENCE

Search: Child* (Topic) OR Adolescen* (Topic) OR Paediatric* (Topic) OR pediatric* (Topic) OR Infant* (Topic) OR Neonat* (Topic)

Result: 2,167211

Search: mortality (Topic) OR morbidity (Topic) OR death (Topic) OR “Child health” (Topic) OR “Disability-adjusted life years” (Topic) OR Anthropometry (Topic) OR Malnutrition (Topic) OR Stunting (Topic) OR overnutrition (Topic) OR Obesity (Topic) OR Disease (Topic) OR disorder (Topic) OR trauma (Topic) OR Anemia (Topic) OR asthma (Topic) OR Respiratory infection (Topic) OR Pneumonia (Topic) OR Diarrhea (Topic) OR Accident (Topic) OR Insecticide-treated net (Topic) OR Immunization* (Topic) OR vaccin* (Topic) OR "Quality of life" (Topic) OR "Childhood cancer " (Topic) OR Hospitalization (Topic) OR Wellbeing (Topic) OR " mental health" (Topic) OR "Health status indicator" (Topic) OR " Life experience " (Topic) and Article or Review Article (Document Types) and Book Chapters (Exclude – Document Types)

Results: 8163283

Search: Refugee (Topic) OR Asylum (Topic) OR Migrant* (Topic) OR "Displaced people camp" (Topic) OR "camp" (Topic)

Result: 82909

Search: #1 AND #2 AND #3 and USA or PEOPLES R CHINA or ENGLAND or AUSTRALIA or CANADA or GERMANY or ITALY or NETHERLANDS or SWEDEN or SPAIN or JAPAN or ISRAEL or SWITZERLAND or TURKEY or FRANCE or NORWAY or BELGIUM or DENMARK or IRELAND or NEW ZEALAND or AUSTRIA or FINLAND or PORTUGAL or GREECE or TURKIYE or TAIWAN or MEXICO or POLAND or SAUDI ARABIA or THAILAND or SINGAPORE or PAKISTAN or RUSSIA or CZECH REPUBLIC or CHILE or NORTH IRELAND or HUNGARY or CROATIA or ROMANIA or U ARAB EMIRATES or VIETNAM or KUWAIT or COTE IVOIRE or URUGUAY or UKRAINE or SRI LANKA or QATAR or ICELAND or ESTONIA or HAITI or PANAMA or FRENCH GUIANA or USSR or TRINIDAD TOBAGO or UNITED KINGDOM or ANDORRA or ST KITTS NEVI or SINT MAARTEN (Countries/Regions) and YUGOSLAVIA or VANUATU or TUNISIA or SURINAME or SOUTH AFRICA or SCOTLAND or BRAZIL or INDIA or JORDAN or LEBANON or WALES or ETHIOPIA or KENYA or UGANDA or IRAN or BANGLADESH or PAKISTAN or TANZANIA or CROATIA or CYPRUS or NEPAL or CAMBODIA or COLOMBIA or EGYPT or GHANA or ARGENTINA or ECUADOR or ESTONIA or NIGERIA or QATAR or LITHUANIA or SRI LANKA or INDONESIA or LUXEMBOURG or MALTA or MOROCCO or PHILIPPINES or SUDAN or BOSNIAHERCEG or DEMREPCONGO or KAZAKHSTAN or MALAWI or NIGER or PERU or RWANDA or SYRIA or AFGHANISTAN or BURUNDI or CAMEROON or EL SALVADOR or IRAQ or LIBYA or MOZAMBIQUE or OMAN or PAPUA N GUINEA or SLOVAKIA or TOGO or ZAMBIA or ZIMBABWE or BOLIVIA or BULGARIA or BURKINA FASO or DOMINICAN REP or ERITREA or FRENCH GUIANA or GEORGIA or GUINEA or JAMAICA or KOSOVO or LIBERIA or MAURITANIA or MONGOLIA or NICARAGUA or NORTH MACEDONIA or SOLOMON ISLANDS or SOMALIA or SURINAME or TUNISIA or VANUATU or YUGOSLAVIA (Exclude – Countries/Regions)

Results: 918
