# Supplementary material for: Health of refugee children upon arrival in high-income countries: A scoping review
Source: J Migr Health. 2025 Oct 29;12:100373. doi: 10.1016/j.jmh.2025.100373 (PMC12664805; doi:10.1016/j.jmh.2025.100373)
Supplement: Supplementary file 3 [file mmc3.docx]

Appendix 3: Critical appraisal using JBI criteria for cross-sectional studies.

| No. | Studies | Criteria (four responses) 1. Yes, 2. No 3. Unclear 4. Not applicable (NA) | | | | | | | | | Decision |
| --- | --- | --- | --- | --- | --- | --- | --- | --- | --- | --- | --- |
|  |  | Were the criteria for inclusion in the sample clearly defined? | Were the study subjects and the setting described in detail? | Was the exposure measured in a valid and reliable way? | Were objective, standard criteria used for the measurement of the condition? | Were confounding factors identified? | Were strategies to deal with confounding factors stated? | Was the condition measured in a standard, reliable way for all participants? | Was appropriate statistical analysis used? | Was the response rate adequate, and if not, was the low response rate managed appropriately? |  |
|  | (Shah et al., 2014) | Yes | Yes | Yes | Yes | NA | NA | Yes | Yes | Yes | Include |
|  | (Newman et al., 2019) | Yes | Yes | Yes | Yes | NA | NA | Yes | Yes | Yes | Include |
|  | (Dawson-Hahn et al., 2016) | Yes | Yes | Yes | Yes | NA | NA | Yes | Yes | Yes | Include |
|  | (Walpole et al., 2018) | Yes | Yes | Yes | Yes | NA | NA | Yes | Yes | Yes | Include |
|  | (Grammatikopoulou et al., 2019) | Yes | Yes | Yes | Yes | NA | NA | Yes | Yes | Yes | Include |
|  | (Heney et al., 2015) | Yes | Yes | Yes | Yes | NA | NA | Yes | Yes | Yes | Include |
|  | (Meyer et al., 2022) | Yes | Yes | Yes | Yes | Yes | No | Yes | Yes | Yes | Include |
|  | (Maldari et al., 2019) | Yes | Yes | Yes | Yes | NA | NA | Yes | Yes | Yes | Include |
|  | (Aucoin et al., 2013) | Yes | Yes | Yes | Yes | NA | NA | Yes | Yes | Yes | Include |
|  | (DeVetten et al., 2017) | Yes | Yes | Yes | Yes | Yes | Yes | Yes | Yes | Yes | Include |
|  | (Beukeboom & Arya, 2018) | Yes | Yes | NA | Yes | NA | NA | Yes | Yes | Yes | Include |
|  | (Scott et al., 2015) | Yes | Yes | Yes | Yes | NA | NA | Yes | Yes | Yes | Include |
|  | (Salehi et al., 2015) | Yes | Yes | Yes | Yes | NA | NA | Yes | Yes | Yes | Include |
|  | (Rungan et al., 2013) | Yes | Yes | Yes | Yes | NA | NA | Yes | Yes | Yes | Include |
|  | (Kloning et al., 2018) | Yes | Yes | Yes | Yes | NA | NA | Yes | Yes | Yes | Include |
|  | (Solberg et al., 2021) | Yes | Yes | Yes | Yes | Yes | Yes | Yes | Yes | Yes | Include |
|  | (Yun et al., 2016) | Yes | Yes | Yes | Yes | NA | NA | Yes | Yes | Yes | Include |
|  | (Gandham et al., 2021) | Yes | Yes | Yes | Yes | NA | NA | Yes | Yes | Yes | Include |
|  | (Solberg et al., 2020) | Yes | Yes | Yes | Yes | Yes | Yes | Yes | Yes | Yes | Include |
|  | (Anil et al., 2022) | Yes | Yes | Yes | Yes | NA | NA | Yes | Yes | Yes | Include |
|  | (Fozouni et al., 2019) | Yes | Yes | Yes | Yes | NA | NA | Yes | Yes | Yes | Include |
|  | (Geltman et al., 2019) | Yes | Yes | Yes | Yes | Yes | Yes | Yes | Yes | Yes | Include |
|  | (Shakya & Bhatta, 2019) | Yes | Yes | Yes | Yes | Yes | Yes | Yes | Yes | Yes | Include |
|  | (Lupone et al., 2020) | Yes | Yes | Yes | Yes | NA | NA | Yes | Yes | Yes | Include |
|  | (Pezzi et al., 2022) | Yes | Yes | Yes | Yes | NA | NA | Yes | Yes | Yes | Include |
|  | (Seifu et al., 2020) | Yes | Yes | yes | yes | No | No | Yes | Yes | Yes | Include |
|  | (Kotey et al., 2018) | Yes | Yes | yes | yes | Yes | Yes | yes | yes | Yes | Include |
|  | (Pavlopoulou et al., 2017) | Yes | Yes | yes | yes | Not clear | Not clear | Yes | Yes | Yes | Include |
|  | (Buchmüller et al., 2018) | Yes | Yes | Not clear | Yes | Yes | Yes | Yes | Not clear | Not clear | Include |
|  | (Riatto et al., 2018) | Yes | Yes | Yes | Yes | NA | NA | Yes | Yes | Yes | Include |
|  | (Calderon & Rominger, 2019) | Yes | Yes | Yes | Yes | NA | NA | Yes | Yes | Yes | Include |
|  | (Theuring et al., 2016) | Yes | Yes | Yes | Yes | NA | NA | Yes | Yes | Yes | Include |
|  | (Macfarlane et al., 2023) | Yes | Yes | Yes | Yes | NA | NA | Yes | Yes | Yes | Include |
|  | (Zwi, Morton, et al., 2017) | Yes | Yes | Yes | Yes | NA | NA | Yes | Yes | Yes | Include |
|  | (Mockenhaupt et al., 2016) | Yes | Yes | Yes | Yes | NA | NA | Yes | Yes | Yes | Include |
|  | (Heudorf et al., 2016) | Yes | Yes | Yes | Yes | NA | NA | Yes | Yes | Yes | Include |
|  | (Janda et al., 2020) | Yes | Yes | Yes | Yes | NA | NA | Yes | Yes | Yes | Include |
|  | (Pezzi et al., 2019) | Yes | Yes | Yes | Yes | Yes | Yes | Yes | Yes | Yes | Include |

Critical Appraisal using JBI criteria for cohort studies.

|  | Studies | Criteria (four responses) 1. Yes, 2. No 3. Unclear 4. Not applicable (NA) | | | | | | | | | | Remark |
| --- | --- | --- | --- | --- | --- | --- | --- | --- | --- | --- | --- | --- |
|  |  | Were the two groups similar and recruited from the same population? | Were the exposures measured similarly to assign people to both exposed and unexposed groups? | Was the exposure measured in a valid and reliable way? | Were confounding factors identified? | Were strategies to deal with confounding factors stated? | Were the groups/participants free of the outcome at the start of the study (or at the moment of exposure)? | Were the outcomes measured in a valid and reliable way? | Was the follow-up time reported and sufficient to be long enough for outcomes to occur? | Was follow-up complete, and if not, were the reasons for loss to follow-up described and explored? | Were strategies to address incomplete follow-up utilised? |  |
|  | (Sandell et al., 2017) | Yes | Yes | Yes | No | Yes | No | Yes | Yes | Yes | No | Include |
|  | (Zwi, Rungan, et al., 2017) | Yes | Yes | yes | No | Yes | Not clear | yes | Yes | yes | No | Include |
|  | (Raymond et al., 2013) | Yes | Yes | Yes | Yes | Yes | Yes | Yes | Yes | Yes | Yes | Include |
|  | (Jensen et al., 2014) | Yes | Yes | Yes | Yes | Yes | Yes | Yes | Yes | Yes | Yes | Include |
|  | (Hanes et al., 2017) | Yes | Yes | Yes | Yes | Yes | NA | Yes | Not clear | Yes | No | Include |
